# Thermally Assisted Atomic-Scale Intermixing and Ordering in GeTe–Sb_2_Te_3_ Superlattices

**DOI:** 10.1021/acsnano.4c13450

**Published:** 2025-02-06

**Authors:** Oana Cojocaru-Mirédin, Jasmin-Clara Bürger, Nikita Polin, Alexander Meledin, Joachim Mayer, Matthias Wuttig, Alwin Daus

**Affiliations:** † I. Institute of Physics (IA), RWTH Aachen University, 52056 Aachen, Germany; ‡ INATECH, University of Freiburg, Emmy-Noether-Straße 2, 79110 Freiburg, Germany; § IMTEK, University of Freiburg, Georges-Köhler-Allee 103, 79110 Freiburg, Germany; ∥ Max-Planck-Institut für Eisenforschung GmbH, 40237 Düsseldorf, Germany; ⊥ Ernst Ruska-Centre (ER-C-2), Forschungszentrum Jülich, 52428 Jülich, Germany; # Jülich-Aachen Research Alliance (JARA-HPC and JARA-FIT), RWTH Aachen University, 52056 Aachen, Germany; ∇ Peter-Grünberg-Institute (PGI 10), Forschungszentrum Jülich, 52428 Jülich, Germany

**Keywords:** superlattice (SL) phase change materials (PCM), chalcogenide superlattice (CSL), layer intermixing, atom probe tomography, metavalent bonding, electrothermal simulation, memory device

## Abstract

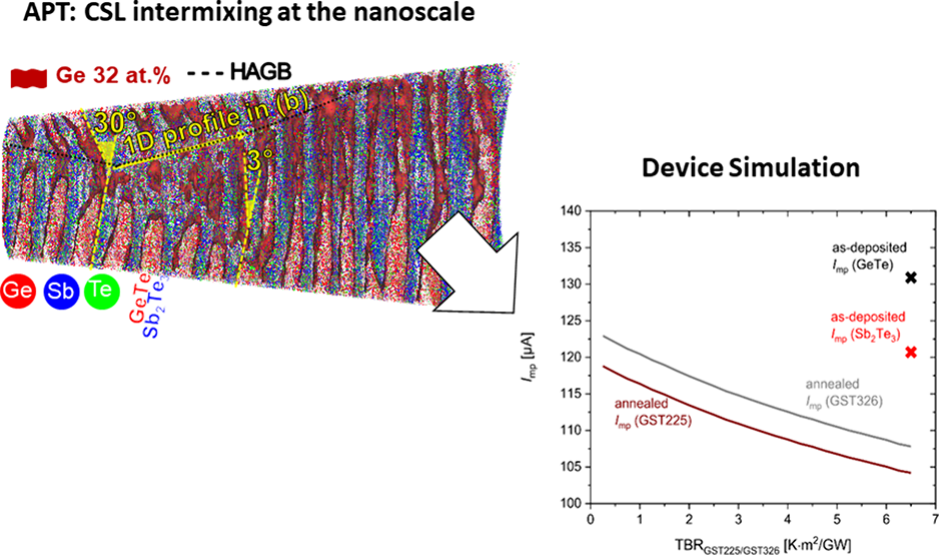

Interfacial phase change memory (iPCM) devices have been shown to switch with significantly reduced power consumption, compared with conventional phase-change memory devices. These iPCMs are based on a periodic structure of nanometer-sized layers of chalcogenides called a chalcogenide superlattice (CSL). Strong temperature increases have been observed within the CSL during the switching procedure, questioning the stability of the CSL structure. In this study, we conduct a detailed quantitative analysis to investigate the evolution of the structure and composition of the sputter-deposited GeTe-Sb_2_Te_3_ CSL upon a temperature increase using atom probe tomography. We find that GeTe-Sb_2_Te_3_ CSLs already feature significant interdiffusion during the synthesis, with a considerable fraction of Sb found in GeTe and Ge in Sb_2_Te_3_. Upon heating the atoms rearrange considerably and form layers of stable Ge_2_Sb_2_Te_5_ and Ge_3_Sb_2_Te_6_ phases, which can be described as a layered solid of GeTe and Sb_2_Te_3_ blocks, i.e., Ge_2_Sb_2_Te_5_ = 2 × GeTe + Sb_2_Te_3_, while Ge_3_Sb_2_Te_6_ = 3 × GeTe + Sb_2_Te_3_. Moreover, these layered solids form in such a way as to preserve and maximize the number of van der Waals (vdW)-like contacts. Interestingly, our electrothermal simulations indicate that the transformation of the original CSL structure into layered stacks of Ge_2_Sb_2_Te_5_ and Ge_3_Sb_2_Te_6_ will have a beneficial effect on device performance. Finally, we discuss the mechanism behind the interdiffusion and phase formation and its implications for iPCM devices. In doing so, the applicability of atom probe tomography to directly investigate intermixing and phase formation on the nanoscale in phase change and related memory devices is demonstrated.

## 1 Introduction

GeTe/Sb_2_Te_3_ chalcogenide superlattices (CSL) have attracted significant interest since their introduction within interfacial phase change memory (iPCM) use as a data storage device. It has been proven that this memory device has a switching speed that exceeds that of conventional bulk phase change memory (PCM) devices (e.g., based on Ge_2_Sb_2_Te_5_)^1,2^), by a factor of ∼3, and provides multibit storage capability with negligible resistance drift as well as improved endurance and reduced power consumption. As the amount of stored data grows exponentially,^3^ reducing the power consumption connected to data storage and processing is an important goal for a sustainable future.

The thermal stability and absence of stoichiometry shifts are of great importance for the functionality of a memory device. These parameters are challenging to handle, because, during the switching process (denoted as SET and RESET processes), Joule’s heat is created in the PCM bulk, resulting in device peak temperatures between 400 and 600 °C.^4^ Moreover, various works found possible stoichiometry shifts due to the atomic motion in PCMs already after a few switching cycles.^5^

Joule’s heat plays a major role in the switching process also for CSL-based memory (or iPCM). Evidence for heat confinement within the superlattice (SL) layer during switching was found recently by Boniardi et al.^6^ and Khan et al.^2^ Furthermore, CSLs are exposed to external heat already during the fabrication, since the growth by magnetron sputtering typically is conducted at elevated temperatures (above 200 °C), potentially causing significant layer intermixing.^7,8^ Thus, understanding this layer intermixing upon a temperature increase becomes crucial for achieving memory devices with an outstanding performance.

Furthermore, several experimental studies exist on structural changes for the heat-treated and switched CSL-based devices using transmission electron microscopy (TEM).^8,9^ However, local changes in the chemical composition of GeTe-Sb_2_Te_3_ CSLs upon heating to different temperatures have not yet been studied. This is mainly because of the difficulty of studying the composition in 3D and down to the atomic level. Therefore, in the present work, the chemical evolution of the CSL GeTe-Sb_2_Te_3_ stack is studied by using atom probe tomography (APT). Moreover, the correlation between composition and structure upon heating is studied utilizing the complementary strengths of APT, TEM, and X-ray diffraction (XRD). XRD is indeed highly beneficial to characterizing the layer structure and structure instabilities directly on the GeTe-Sb_2_Te_3_ superlattices, without tedious sample preparation.

On the other hand, the CSL structure including layer stoichiometries and the existence of van der Waals (vdW)-like gaps will influence the overall thermal and electrical properties of the stack,^10,11^ which govern the generation of Joule’s heat during device switching. In this context, we have recently shown that the current needed to reach the material melting point can be estimated through numerical electrothermal simulations.^2^ We note here that multiple theories on the switching mechanism in CSL exist,^6^ but in our view, the thermal-based melt-quench process is sufficiently well supported by recent literature and the electrothermal simulations adequately describe the device switching currents obtained in devices.^2,6^ Thus, the structural changes in the CSL after annealing, affecting the thermal and electrical resistances in the device, will alter the Joule heating efficiency and, therefore, the switching characteristics.

Hence, in this work, we present a nanoscopic scenario for the intermixing that takes place between Sb_2_Te_3_ and GeTe CSL layers and causes the formation of Ge_2_Sb_2_Te_5_ and Ge_3_Sb_2_Te_6_ lamellae upon heating at a temperature of 350 °C for 30 min. Interestingly, these two phases persist even when the heat treatment is prolonged for 24 h proving their thermal stability. Moreover, we demonstrate that the strongest intermixing between Sb_2_Te_3_ and GeTe CSL layers takes place at the high-angle grain boundaries (GBs) in agreement with recent work.^12^ These structural defects are considered to be preferable sites for the heterogeneous nucleation of Ge_2_Sb_2_Te_5_ and Ge_3_Sb_2_Te_6_.

Last, but not least, to guarantee the functionality of CSL-based devices, it is essential that the CSL can melt in the as-deposited state and also after annealing. This requires analysis of the temperatures developed in the device. Since the measurement of the temperature development in the CSL is difficult during the device operations, finite-element-method (FEM) simulations are a method of choice.^2^ Simulations have shown a major temperature increase in the CSL structure in the literature.^2,13^ However, the focus was mainly laid on the as-deposited GeTe/Sb_2_Te_3_ CSL structures.^2^ The temperature changes developed over time or after an annealing step were often not investigated. Since these structures are used for memory devices, long-term functionality must be maintained; hence, we have explored the development of the temperature during operation.

This includes not only the one SET and RESET process cycle for permanent data storage similar to compact discs (CDs)^14^ but also repeated switching processes as the CSL-based memory must compete with memory technologies as, e.g., the dynamic random-access memory (DRAM) devices or flash.^15^ Based on our experimental results, we conduct FEM simulations before and after annealing for analysis of CSL-based memory devices. This not only provides information about the impact of an annealing step during device fabrication but also can be used as an indication of device lifetime after a high number of switching cycles. Concerning a detailed discussion on the different material properties of GeTe, Sb_2_Te_3_, Ge_2_Sb_2_Te_5_, and Ge_3_Sb_2_Te_6_, we investigate the influence of the experimentally observed intermixing on the device performance and its long-term stability.

## 2 Results and Discussion

### 2.1 CSL Bulk Structure Evolution down to the Nanoscale

**Figure 1:**
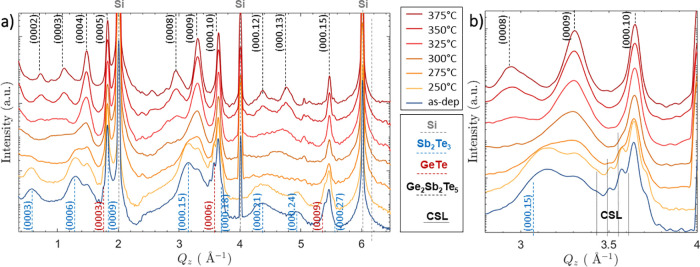
XRD-T2T investigations of ∼220 nm thick Sb_2_Te_3_–GeTe superlattices sputter-deposited on Si(111) substrate. (a) T2T symmetrical XRD scan of the CSL films and (b) zoomed-in scan region around the second superlattice peak group around *Q*_*z*_ = 3.6 Å^–1^. Vertical dashed lines show the (*lll*)-peak family for the Si(111) substrate and the (000*l*)-peak families for Sb_2_Te_3_, GeTe, and Ge_2_Sb_2_Te_5_. SL 4 peaks group in gray in panel (b) are found around positions where peaks of GeTe and Sb_2_Te_3_ are close to each other. At 300 °C, the supercell periodicity is lost, and at 350 °C, the stable Ge_2_Sb_2_Te_5_ phase forms. XRD measurements suggest a transformation of the Sb_2_Te_3_–GeTe CSL into a stable Ge_2_Sb_2_Te_5_ phase upon heat treatment.

Figure 1 shows XRD-Theta-2Theta (XRD-T2T) measurements of deposited and annealed CSLs at temperatures between 250 and 375 °C. For better visual comparison the curves are separated by an offset. The XRD-T2T scans in Figure 1 give information about periodicities in the out-of-plane direction, i.e., the thin film growth direction. Peaks at *Q*_*z*_ of 2 Å^–1^, 4 Å^–1^, and 6 Å^–1^ stem from the Si(111) substrate and are of no further interest in this work.

CSL peak groups at *Q*_*z*_ = 1.8, 3.6, and 5.4 Å^–1^, which consist of a main peak and satellite peaks (i.e., equidistantly spaced peaks with small reciprocal spacing Δ*Q*_*z*_ around the main peak, as shown in Figure 1b for a zoomed-in view), are visible for the as-deposited sample. These superlattice peak groups evolve around the main peak reflexes of Sb_2_Te_3_ and GeTe such that the main peak encodes the mean Te–Te-distance of both materials.^16^ Satellite peaks stem from larger real space periodicity than atomic distances, i.e., from the unit cell size of the superlattice (supercell), which is a double layer of GeTe and Sb_2_Te_3_. From the spacing Δ*Q*_*z*_ of these superlattice peaks, the supercell size Λ = 8.9 nm can be determined, according to Λ = 2π/Δ*Q*_*z*_. The fitting procedure is described by Hollermann et al.^16^ Moreover, additional peaks are found at *Q*_*z*_ = 0.62 Å^–1^, which can be attributed to the (0003) family of Sb_2_Te_3_, i.e., scattering at quintuple layers (QL) of Sb_2_Te_3_.

These CSL peak groups start to diminish when the sample is annealed at temperatures between 250 and 300 °C and vanish completely at temperatures above 300 °C, as shown in Figure 1b. Interestingly, at 300 °C, the Ge_2_Sb_2_Te_5_ peaks emerge, indicating that the CSL has reconfigured into Ge_2_Sb_2_Te_5_ ((0001) family of Ge_2_Sb_2_Te_5_: GST225) which has an out-of-plane periodicity of 9-tuples. The Ge_2_Sb_2_Te_5_ phase remains stable with increasing annealing temperature until 375 °C. Quantitative evaluation of the XRD data gives lattice constant *c*_GST225_ of 17.23 ± 0.12 Å (see Figure S1(b) in the Supporting Information), which is consistent with the literature.^12^

As determined by XRD, only the predominant atomic arrangement of the CSLs can be measured; further measurements are needed to reveal if other phases than Ge_2_Sb_2_Te_5_ are formed at 350 °C, too. For that, the structure of the CSL sample, which was annealed at 350 °C, is investigated down to the nanoscale using TEM.

**Figure 2:**
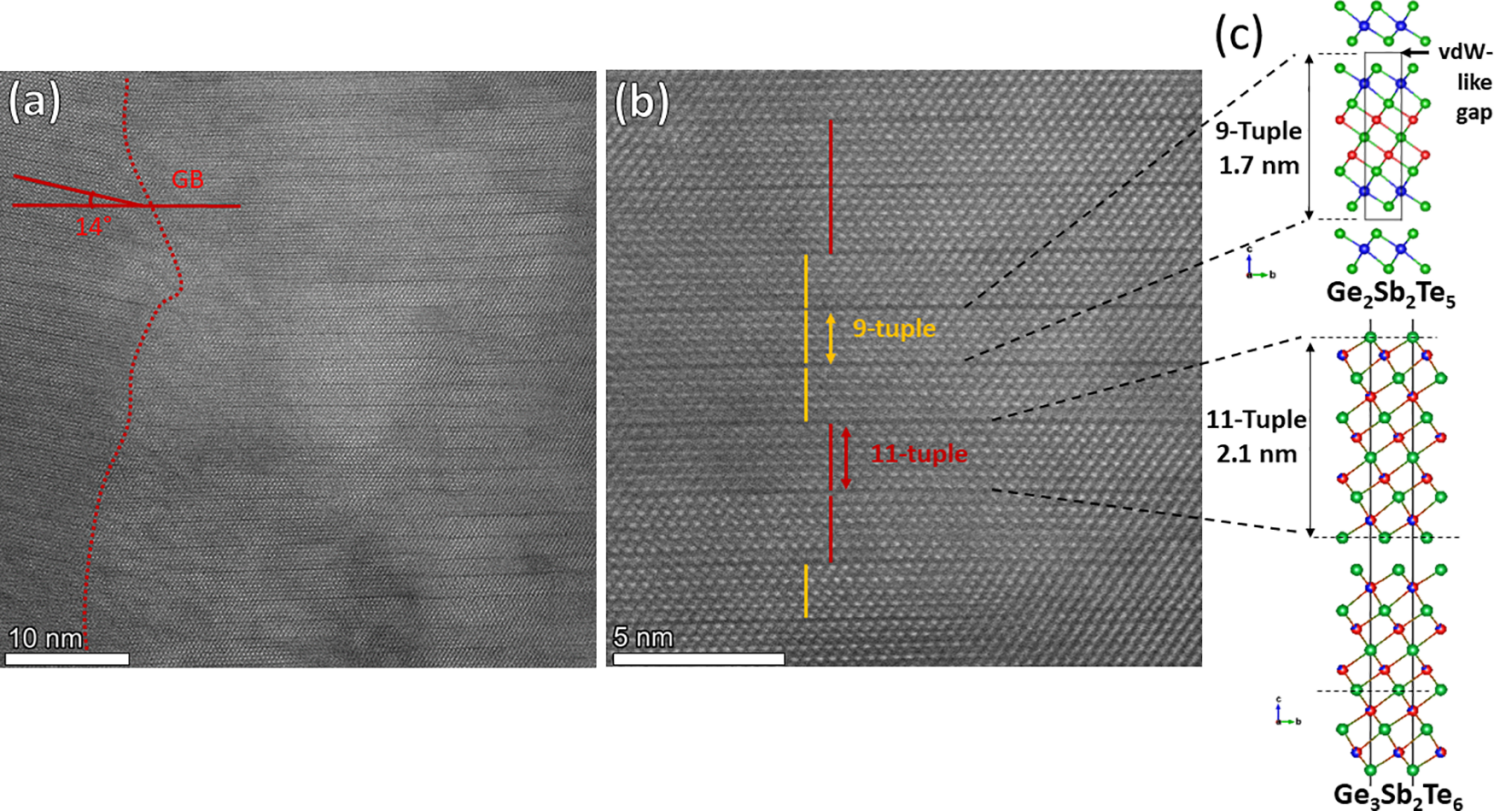
STEM investigations of the annealed Sb_2_Te_3_–GeTe superlattices together with corresponding crystal structures. (a) and (b) HAADF STEM micrographs of CSL layer sputter deposited on Si(111) substrate and annealed at 350 °C observed at different magnifications. (c) Additionally, corresponding crystal structures for Ge_2_Sb_2_Te_5_ and Ge_3_Sb_2_Te_6_ are displayed. The STEM measurements show that the initial CSL structure with the expected phases of Sb_2_Te_3_ and GeTe as present in the as-deposited state (see Figure S2 in the Supporting Information) transforms to a stacked structure with the coexistence of Ge_2_Sb_2_Te_5_ (9-tuple) and Ge_3_Sb_2_Te_6_ (11-tuple) phases.

Figure 2 shows high-angle annular dark-field scanning TEM (HAADF-STEM) micrographs of a 350 °C annealed sample. As can be seen, “vdW-like” gaps are present throughout the entire sample, highlighting the layered structure. Figure 2b shows a magnified region of the right grain in Figure 2a. Interestingly, this region locally shows two types of tuples: 9-tuples and 11-tuples. If the 9-tuples correspond to the expected Ge_2_Sb_2_Te_5_ phase from the above XRD investigation, then the 11-tuples correspond to the Ge_3_Sb_2_Te_6_ phase. Hence, these STEM measurements suggest that the initial CSL structure with Sb_2_Te_3_ and GeTe phases in the as-deposited state (see Figure S2 in the Supporting Information) transforms to a stacked structure with the coexistence of Ge_2_Sb_2_Te_5_ and Ge_3_Sb_2_Te_6_ phases. It might seem surprising, at first glance, that GeTe and Sb_2_Te_3_ transform to Ge_2_Sb_2_Te_5_ and Ge_3_Sb_2_Te_6_. But it is indeed expected that Ge_2_Sb_2_Te_5_ forms when one layer of Sb_2_Te_3_ is mixed with two layers of GeTe, while Ge_3_Sb_2_Te_6_ rather forms when the Sb_2_Te_3_ compound is not available in a sufficient quantity, i.e., when one layer of Sb_2_Te_3_ is mixed with three layers of GeTe. While this can be shown also from thermodynamic data and the corresponding phase diagram, we still need to understand why Ge_2_Sb_2_Te_5_ and Ge_3_Sb_2_Te_6_ form as a layered stack. A similar ordering would not be expected and is not observed if two vdW-bonded solids such as MoS_2_ and WS_2_ are mixed. In this case, a solid solution of the two solids, i.e., MoS_2_ and WS_2_, is found. In contrast, for Ge_2_Sb_2_Te_5_ and Ge_3_Sb_2_Te_6_, this is not the case; instead, an ordered and layered stack of Ge_2_Sb_2_Te_5_ and Ge_3_Sb_2_Te_6_ is formed. The reason for this stacking is the strong coupling across the different building blocks, i.e., GeTe and Sb_2_Te_3_, which even explains the diversity of similar compounds in the Earth’s crust.^17^

Additionally, a low-angle grain boundary (LAGB) with a disorientation angle of 14° is observed, as shown in Figure 2a. At the grain boundary (GB), some of the “vdW-like” gaps are interrupted while others are maintained throughout the LAGB and are, thus, kinked (cf. Figure S3(a) in the Supporting Information). Moreover, the region around this GB shows not only the presence of 9- and 11-tuples but also of 7-tuples, which corresponds to the Ge_1_Sb_2_Te_4_ phase (cf. Figure S3(a) in the Supporting Information). Additionally, the existence of 7-tuples was also confirmed in a different region at the bottom of the CSL layer, close to the Si (111) substrate, as shown in Figure S3(b). Moreover, the formation of Ge_3_Sb_2_Te_6_ and Ge_1_Sb_2_Te_4_ phases at 350 °C was invisible in our XRD investigations, suggesting that there are found below the limit of measurement accuracy.

### 2.2 CSL Composition Evolution down to the Nanoscale

Although STEM investigations already showed the nature of the phases formed upon annealing, it remains unclear how the CSL composition evolves. This is because of the difficulty in determining the degree of intermixing among the CSL layers upon annealing. Therefore, we applied the APT capabilities to study the composition evolution of the CSL layer upon annealing.

**Figure 3:**
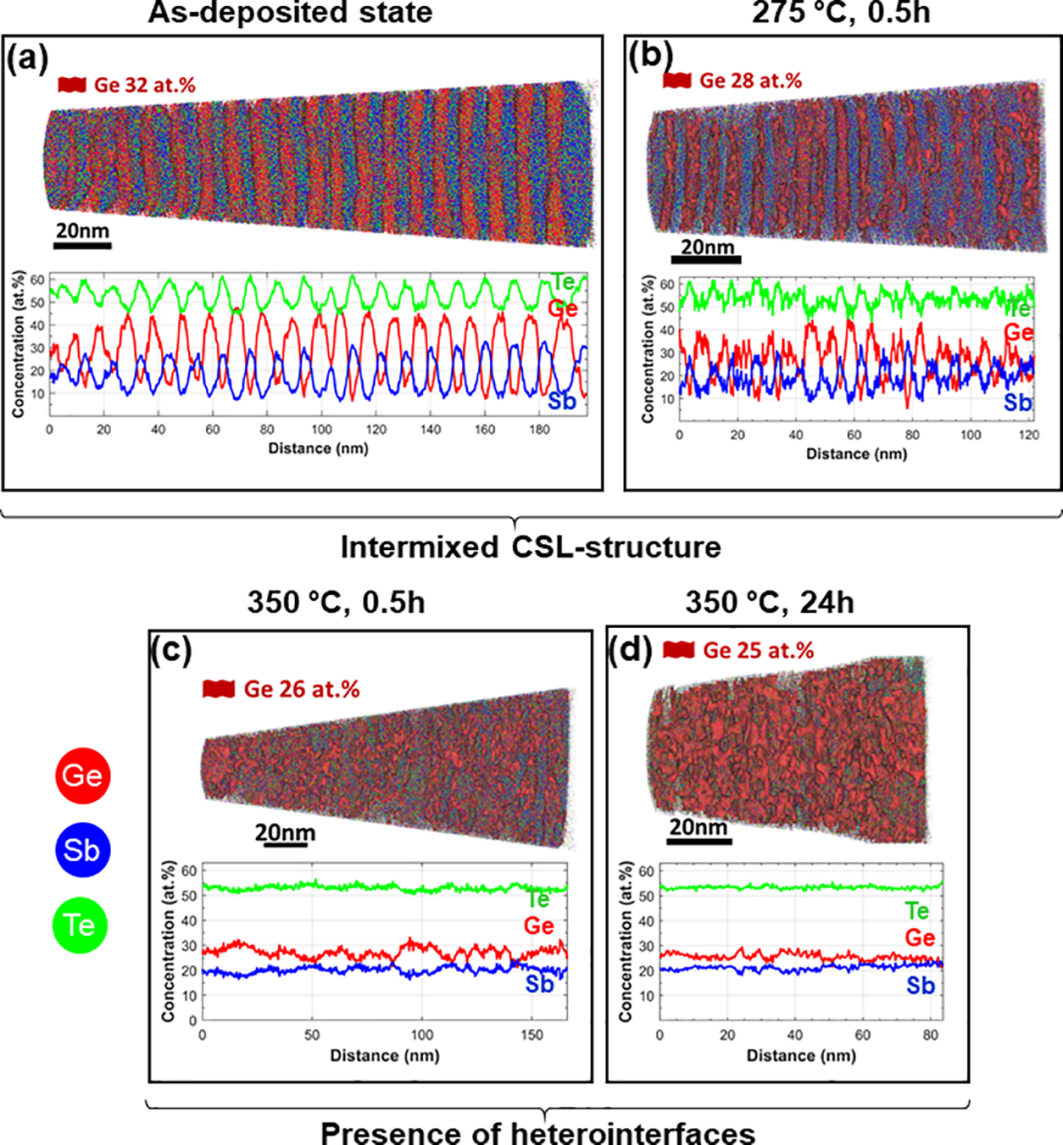
Summary of the APT investigations showing the 3D elemental maps and corresponding 1D concentration profiles of Ge (red), Sb (blue), and Te (green). The samples investigated are as-deposited, 275 °C–0.5 h, 350 °C–0.5 h, and 350 °C–24 h. The interfaces are marked by 25–32 at. % Ge isosurfaces as marked for each sample. The 1D concentration profiles are calculated along elongated probing volumes oriented in the growth direction. The probing volumes are sampling boxes or cylinders with cross sections between 10 nm × 10 nm to 40 nm × 40 nm with different lengths, depending on the size of the APT data. These APT measurements show the intermixing evolution upon heat treatment: Initially intermixed CSL layers evolve into well-discernible Ge_3_Sb_2_Te_6_ and Ge_2_Sb_2_Te_5_ lamellae. Note that these phases are stable, because they are still present in the CSL layer annealed at 350 °C for a comparably long annealing duration of 24 h.

Figure 3 summarizes the APT investigations of the CSL layer (see the Experimental Section for more information about CSL preparation) for the as-deposited state as well as after different annealing steps (275 °C–0.5 h, 350 °C–0.5 h, and 350 °C–24 h). The 3D maps for the as-deposited and 275 °C–0.5 h state show an intermixed CSL structure where the GeTe and Sb_2_Te_3_ sequences are still distinguishable, whereas the 3D maps of 350 °C–0.5 h and 350 °C–24 h states exhibit lamellar regions separated through heterointerfaces. For the last two states, no GeTe and Sb_2_Te_3_ layers are available, which agrees with the XRD and TEM results.

**Figure 4:**
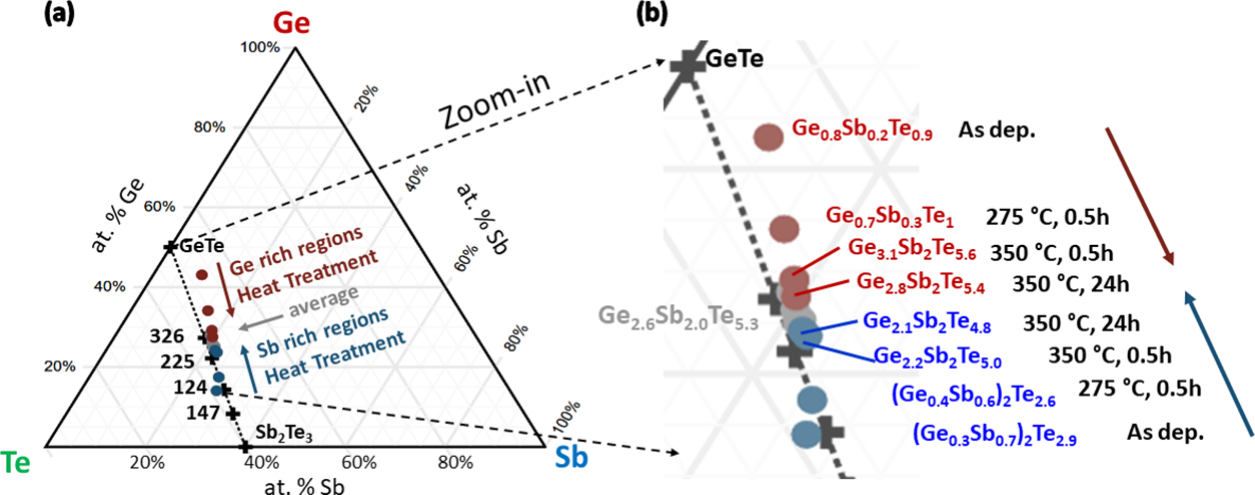
(a) Complete and (b) zoomed-in version of the Ge–Te–Sb ternary phase diagram. Between the expected two stable phases (GeTe and Sb_2_Te_3_), several Ge-rich and Sb-rich nonstable compounds are available. These compounds were extracted and averaged from the APT 1D concentration profiles of the CSLs. The known Ge_*x*_Sb_*y*_Te_*z*_ compounds are marked by black crosses with corresponding formula coefficients, i.e., “326” stands for Ge_3_Sb_2_Te_6_.

The corresponding 1D concentration profile of the as-deposited state shows that chemical intermixing between GeTe and Sb_2_Te_3_ already occurs during deposition at 200 °C with ∼10 at. % Sb found in GeTe and ∼13 at. % Ge found in Sb_2_Te_3_, in agreement with our recent work.^7^ At 275 °C, this intermixing gets even stronger such that in certain places the CSL structure vanishes. The distinguishable CSL layers are even more intermixed with (11 ± 2) at. % Sb in GeTe and (14 ± 5) at. % Ge in Sb_2_Te_3_, confirming the high Sb solubility in GeTe and of Ge in Sb_2_Te_3_. This pronounced intermixing has already been observed for thermoelectrics such as (GeTe)_*x*_(AgSbTe_2_)_1–*x*_ (TAGS-*x*)^18^ or (Ge_0.84_Sb_0.06_Te_0.9_)(30 PbSe)_0.05_(PbS)_0.05_.^19^ For those solids, pronounced intermixing of dopants was observed, if the dopant could be incorporated into the metavalent crystal.^20−22^ The composition of the layers can be written as Ge_0.8_Sb_0.2_Te_0.9_ for the original GeTe layer or Ge_0.7_Sb_1.3_Te_2.8_ or Ge_1.0_Sb_2_Te_4.1_ for the Sb_2_Te_3_ layer (see Figure 4). However, at 350 °C, no CSL layer is visible in the 1D concentration profile anymore. Instead, two lamellar structures (i.e., Ge-rich lamellae with ∼31 at. % Ge and Ge-poor lamellae with ∼22 at. % Ge) are found. The composition of these layers can be written as Ge_3.5_Sb_2.0_Te_5.8_ for the Ge-rich lamellae and Ge_2.0_Sb_2.0_Te_4.8_ for the Ge-poor environment. That means that at 350 °C the composition fluctuates between the Ge_2_Sb_2_Te_5_ and Ge_3_Sb_2_Te_6_. Moreover, the overall bulk composition of the CSLs (normalized to *y* = 2 in Ge_*x*_Sb_*y*_Te_*z*_ given that the samples were slightly Te deficient due to the Te volatility during the sputter deposition) derived from all regions investigated for the sample 350 °C is Ge_2.58_Sb_2_Te_5.27_ which lies in between the stable Ge_2_Sb_2_Te_5_ and Ge_3_Sb_2_Te_6_ compounds, as detailed in Figure S4 in the Supporting Information.

Figure 4a shows the Ge–Sb–Te ternary phase diagram, where the composition evolution is given for each heat treatment applied. In the as-deposited state, the compositions deviate significantly from the stoichiometric GeTe and Sb_2_Te_3_ because a strong intermixing between these two phases takes place, as described above. Even though the composition deviates strongly from GeTe or Sb_2_Te_3_ respectively, the structure is preserved as proven above by the XRD and TEM investigations. Upon heat treatment, the compositions approach each other, which indicates that the intermixing between GeTe and Sb_2_Te_3_ becomes even more distinct (see the zoomed-in region of the ternary phase diagram in Figure 4b). However, they do not coincide and remain closest to Ge_2_Sb_2_Te_5_ and Ge_3_Sb_2_Te_6_, even after applying long annealing durations, such as 24 h at 350 °C.

**Figure 5:**
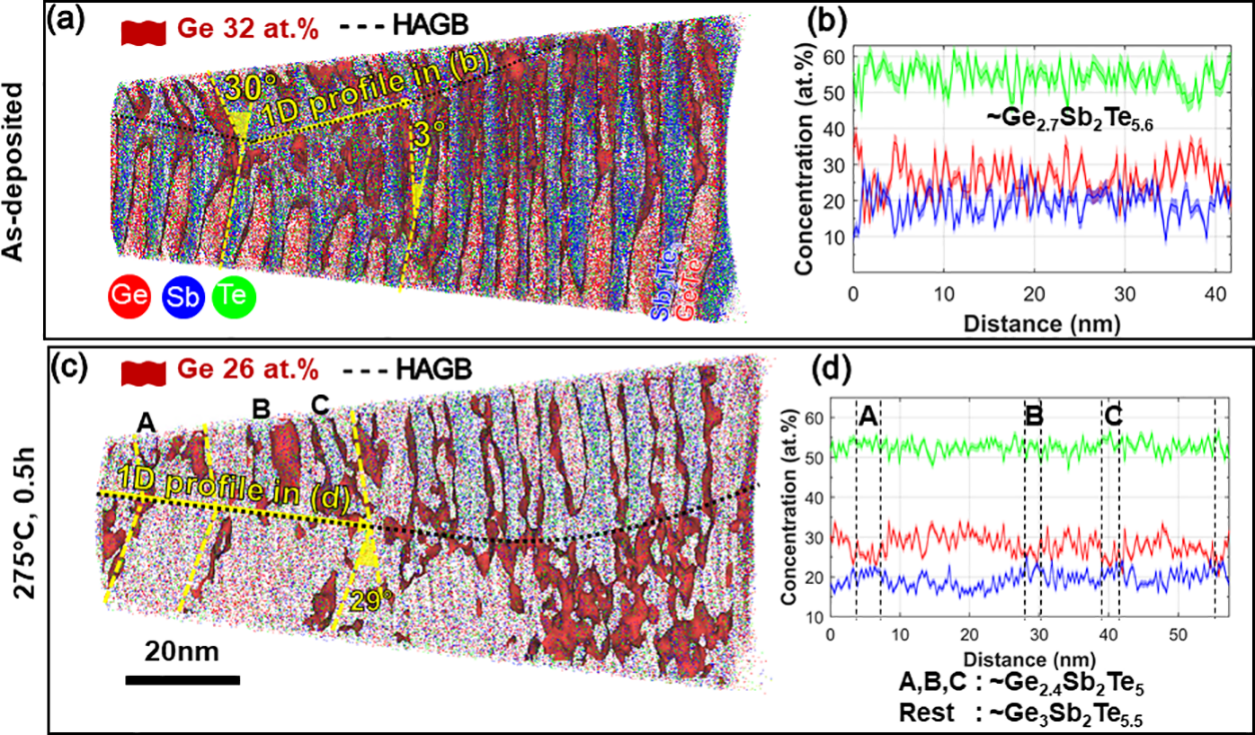
APT composition investigations of high angle grain boundaries (HAGBs). Here, two representative areas were extracted from the as-deposited sample and a sample annealed at 275 °C for 0.5h. APT 3D elemental maps in panels (a) and (c) as well as the corresponding 1D concentration profiles of Ge (red), Sb (blue), and Te (green) in panels (b) and (d) were calculated along the grain boundaries (highlighted by black dotted lines) in as-deposited and 275 °C–0.5 h samples, indicated by yellow arrows and labeled “1D profile in (b)/(d)”. The 1D concentration profiles were built using a sampling box of 5 nm × 5 nm × 0.2 nm and 8 nm × 10 nm × 0.2 nm for as-deposited and 275 °C–0.5 h samples, respectively. The grain boundary composition in 275 °C–0.5 h approaches the compositions of Ge_3_Sb_2_Te_6_ and Ge_2_Sb_2_Te_5_. APT measurements show increased intermixing at the grain boundaries, compared to the bulk phase.

Figure 5 summarizes some representative high-angle grain boundaries (HAGBs) with disorientation angles of 29°–30° measured for the as-deposited and 275 °C–0.5 h samples. Interestingly, strong intermixing is observed at the GBs, i.e., much more pronounced than that in the bulk region. For example, the composition of the HAGB in the as-deposited sample approaches astonishingly already the composition of the Ge_3_Sb_2_Te_6_ phase (see Figure 5b), although the bulk contains a layered structure with Ge-doped Sb_2_Te_3_ and Sb-doped GeTe SL. Even more, the HAGB composition approaches the composition of Ge_3_Sb_2_Te_6_ and Ge_2_Sb_2_Te_5_ phases already at 275 °C, even though these phases were present in the CSL bulk only at 350 °C. This suggests that the HAGBs facilitate the compositional changes that take place in the bulk at higher temperatures by enabling an efficient interdiffusion path. This is due to the much-stronger intermixing effects that occur at the GBs than inside the bulk CSL bulk. This phenomenon is well-known and has been attributed to the lower activation energy for interdiffusion at grain boundaries, compared with the bulk.

### 2.3 Layer Intermixing in CSL

**Figure 6:**
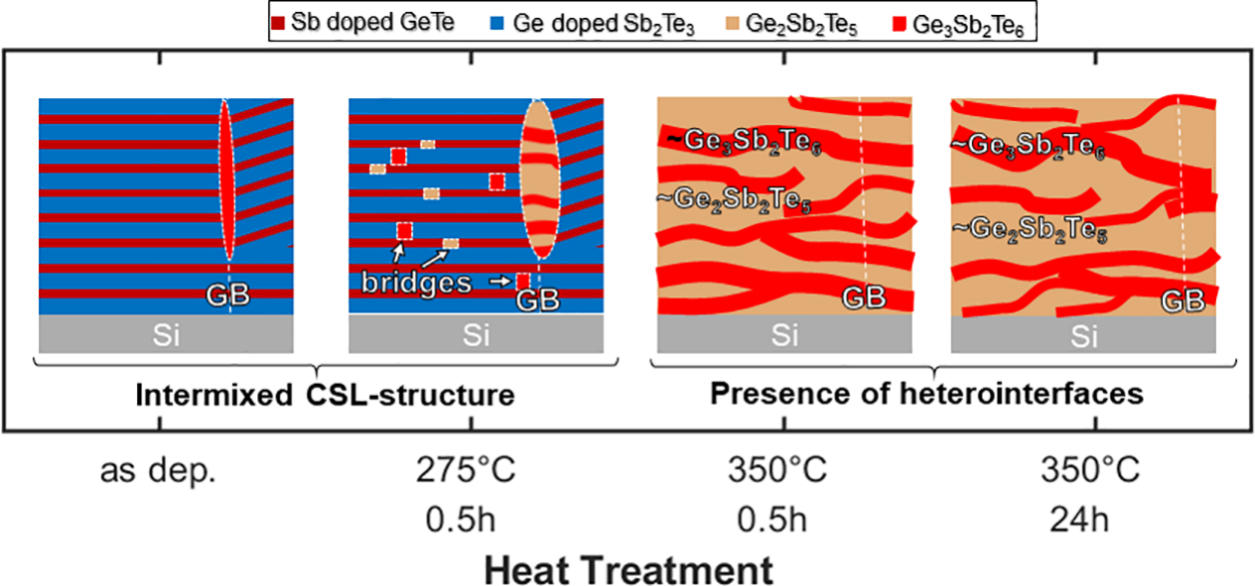
Schematic representation of the intermixing increase in CSLs upon heat treatment based on the APT/TEM/XRD results. Initially, CSL with GeTe and Sb_2_Te_3_ layers in the as-deposited state turn into Ge_3_Sb_2_Te_6_ and Ge_2_Sb_2_Te_5_ lamellar structure upon heat treatment at 350 °C. The intermixing proceeds faster at grain boundaries and so-called “bridges”, where Ge_3_Sb_2_Te_6_ and Ge_2_Sb_2_Te_5_ precipitates form already at an earlier stage of heat treatment, such as 275 °C.

Figure 6a schematically depicts the findings of the present work based on XRD, TEM, and APT investigations. Initially, the CSL consists of Ge-doped Sb_2_Te_3_ and Sb-doped GeTe layers (cf. Figure 3a). Despite the significant composition deviation of the layers (∼10 at. % Sb in GeTe and ∼13 at. % Ge in Sb_2_Te_3_, as shown in Figure S4), the stacking of the layers is preserved as confirmed by XRD and TEM. Upon annealing at 275 °C, the GeTe and Sb_2_Te_3_ layers partially rearrange, such that bridges with Ge_3_Sb_2_Te_6_ and Ge_2_Sb_2_Te_5_ stoichiometry are formed between two neighboring GeTe or Sb_2_Te_3_ layers, as demonstrated in Figure S5 in the Supporting Information. Further annealing to 350 °C turns the superlattice into a lamellar structure consisting of Ge_3_Sb_2_Te_6_ and Ge_2_Sb_2_Te_5_. The Ge_3_Sb_2_Te_6_ phase could not be identified by XRD, but only by TEM and APT. Interestingly, the Ge_3_Sb_2_Te_6_ and Ge_2_Sb_2_Te_5_ phases with a lamellar structure persist even after the 24-h long heat treatment at 350 °C, where these phases undergo only small stoichiometric shifts (<2 at. %). This finding implies that the corresponding layer stacking is energetically more favorable.

A potential explanation for such strong intermixing between GeTe and Sb_2_Te_3_ layers is provided by Kooi et al.^9^ They observed a reordering from CSL structure into stable GST due to the “vdW reconfiguration” process. This consists of the Sb–Te displacement along the *c*-axis and, therefore, also the displacement of the vdW-like gap.^9^ Yet, at higher temperatures, Kooi et al.^9^ observed only the GeSb_2_Te_4_ phase; i.e., only 7-tuples by STEM and an homogeneous composition by EDX. Their results do not coincide with the present results where the Ge_3_Sb_2_Te_6_ and Ge_2_Sb_2_Te_5_ phases have been observed. The main reason for this difference is that, contrary to Kooi et al.’s CSL,^9^ which were GeSb_2_Te_4_-stoichiometric, the CSLs in this work show an off-stoichiometry Ge_2.6_Sb_2.0_Te_5.3_, which is between Ge_3_Sb_2_Te_6_ and Ge_2_Sb_2_Te_5_ (see Figure 5). This highlights the importance of the initial CSL stoichiometry on the phase formation during heat treatment, or, in a larger context, during switching processes. The overall CSL stoichiometry is determined by the ratio of the initial layer thickness of Sb_2_Te_3_ to GeTe. In this work, we employ CSL based on a sequence of 5 nm Sb_2_Te_3_ and 4 nm GeTe, while, in the work of Kooi et al.,^9^ the CSL sequence contains 3 nm Sb_2_Te_3_ and 1 nm GeTe.

The pseudobinary phase diagram (see Figure S6 in the Supporting Information) predicts stable phases of GeTe and Sb_2_Te_3_ for only a small amount of impurities below 2 at. %. This implies that the highly doped GeTe and Sb_2_Te_3_ phases in this work are in a metastable state rather than in a stable state. An intuitive formulation is elemental diffusion due to Fick’s law, where the concentration gradient  for each of the elements Ge, Sb, Te is the driving force .^23^ Here, Ge and Sb diffuse faster than Te, due to higher concentration gradients.

The Ge_*x*_Sb_*y*_Te_*z*_ compounds are characterized by a high vacancy concentration of ∼10%, due to their low formation energies when sitting on the Ge/Sb sublattice, in striking contrast to typical semiconductor materials such as Si and GaAs, where vacancy formation energies are large.^24,25^ Moreover, two types of cation vacancies have been identified in Ge_*x*_Sb_*y*_Te_*z*_ compounds: the nonstoichiometric excess vacancies and the stoichiometric vacancies. The high hole concentration in crystalline GeTe and GeSb_2_Te_4_ of ∼5 · 10^20^ cm^–3^ and 0.8 · 10^20^ cm^–3^ is due to the presence of excess Ge and/or Sb vacancies.^26−28^ These defects produce a *p*-type metallic conduction by pushing the Fermi level into the valence band. In contrast, the stoichiometric vacancies do not contribute to the formation of charge carriers. Nonetheless, they affect strongly the electrical and thermal transport properties of the material.^29,30^ These stoichiometric vacancies are needed to reach an average of 3 p-electrons per site (for example, for the GeSb_2_Te_4_ compound, 25% stoichiometric vacancies are necessary on the cation sublattice), which implies that, for GeTe (Ge:3d^10^4s^2^4p^2^ and Te: 4d^10^5s^2^5p^4^), no such stoichiometric vacancies are needed.^31^ Interestingly, Zhang et al.^31^ had shown that compounds such as Ge_2_Sb_2_Te_5_ and Ge_3_Sb_2_Te_6_ contain vacancy clusters that prevent the charge carriers from contributing to transport and render these compounds insulating. Yet, these vacancy clusters are known to dissolve at high annealing temperatures turning the system back to the metallic state through metal-to-insulator transition (MIT).^32^

There is no doubt that the presence of these stoichiometric vacancies at low temperatures will induce high disorder in the system. Besides the concentration gradient , mentioned above as a driving force for intermixing, another driving force for the intermixing can be the minimization of the free energy *F* of the CSL system by internal energy maximization,
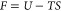
where *U* is the internal energy, *T* the temperature, and *S* the entropy of the CSL. This internal energy minimization is due to the “bonding hierarchy”, i.e., when mixing Sb_2_Te_3_ and GeTe the structure reconfigures such that the vdW-like gaps are preserved. That means that the ordering into GST225 and GST326 is energetically driven. These vdW-like Te–Te bonds present initially in Sb_2_Te_3_ but not in GeTe. We note here that the term “vdW-like” is possibly misleading. These solids are characterized by a significantly stronger interaction than the typical vdW forces that prevail in most transition-metal dichalcogenides (TMDCs), boron nitride (BN), and graphene. In fact, these bonds are present in many other compounds such as Bi_2_Te_3_ and Bi_2_Se_3_ and have a reduced length of 16.2%, when compared with typical vdW gaps (3.1 ± 0.03 Å, compared to 3.7 Å), leading to non-negligible electron sharing in the gaps.^11^ Moreover, the materials containing these bonds have unconventional properties that are crucial for PCM devices such as high dielectric constant and high electronic conductivity.^33^ One general observation made throughout this and previous studies is that the vdW-like gaps are preserved during intermixing between GeTe (no vdW-like gaps) and Sb_2_Te_3_ (with vdW-like gaps) even at temperatures of 600 °C.^2^ Therefore, one possible explanation for the coexistence of Ge_3_Sb_2_Te_6_ and Ge_2_Sb_2_Te_5_ lamellae is the preservation of these vdW-like gaps. These vdW-like gaps or bonds are weaker than the covalent bonds but much stronger than the classic vdW bonds, as already emphasized above and in ref (17).

Another very intriguing finding in this work is the strong intermixing capability between Sb_2_Te_3_ and GeTe already for the as-deposited state, while the structure is well-preserved. Specifically, a high quantity of Ge is found in Sb_2_Te_3_ and the opposite. In fact, both materials, Sb_2_Te_3_ and GeTe, are metavalent-bonded (MVB) materials, which have been very recently discovered to host a high quantity of dopants.^19^ The reason for this significant dopant tolerance is the weak and flexible metavalent bond that these solids employ.^34^

Last but not least, compared to the CSL bulk, the high-angle GBs are high-energy structural defects.^35^ Moreover, it is well-known that these structural defects host a higher density of point defects such as vacancies and interstitials, as well as dangling bonds. These factors lead to higher intermixing rates at the GBs compared with those in the bulk (or grain interiors). This explains why the Ge_3_Sb_2_Te_6_ and Ge_2_Sb_2_Te_5_ phases (present in the CSL bulk only at 350 °C) are detected at the GBs of the CSL thin film already at 275 °C and even for the as-deposited state. Hence, the GBs provide a path for phase formation in the bulk. It is important to mention here that no such heterogeneous nucleation of GST-based phases at grain boundaries was reported in the literature due to the difficulty of investigating the composition in 3D and down to the subnanometer level.

### 2.4 Impact of Layer Intermixing on iPCM Devices

Figures 2 and 3 show that a thermal treatment of 350 °C for 24 h led to the intermixing of the GeTe-Sb_2_Te_3_ CSL to form a Ge_2_Sb_2_Te_5_ and Ge_3_Sb_2_Te_6_ SL. The formation of GeSbTe alloys upon thermal treatment has been indeed shown previously,^36^ but it was not mentioned exactly that GST225 and GST326 are formed.

The annealing can be regarded as an accelerated aging experiment for CSL materials, which allows us to predict long-term changes or thermal cycling effects, e.g., after multiple switching processes. Previous studies have shown that the temperatures in the CSL-based memory are in the range of the investigated annealing temperature of 350 °C and above.^2^ In fact, a part of the device will reach the material melting point of ∼600 °C during the so-called melt-quench process.^37,38^ Even though this temperature is higher than the 350 °C of annealing conducted in this study, the melt-quench process is operated in nanoseconds, limiting the intermixing time scale of each switching (RESET) event. Thus, 24 h of annealing conducted here can represent a settled material reconfiguration situation of a well-cycled PCM device.

We now investigate the as-deposited and annealed CSL structures by using electro-thermal simulations with COMSOL and we compare them with the APT results. This represents the first kind of correlative APT-COMSOL simulation study to understand whether the required current pulses to reach the device RESET (melting point for melt-quench process) are expected to change upon the structural reconfiguration of the CSL. We simulated a configuration as it could be found for a monolithically integrated CSL device on a complementary metal-oxide semiconductor (CMOS) chip.^39−41^ It was assumed that the memory layer would be integrated into the upper layers of a CMOS chip and fully integrated into a SiO_2_ matrix. The design is based on standard materials and geometries in standard CMOS technology.^39−41^ The thicknesses of the PCM layers are based on the experiment design, as described in Experimental Section and Figure 2. Furthermore, to improve the informative value of the simulation and its consistency with the experiment, the input values for the simulation (i.e., thermal and electrical conductivity values) were based on experimental data from CSLs fabricated with the same method. Using such parameters directly includes the impact of vdW-like gaps within the layers. Please refer to Table S1 in the Supporting Information and the simulation section in the paper for further information. By this, the comparability of the experimental results of this paper with the simulations of the GeTe-Sb_2_Te_3_ and GST225–GST326 memory devices should be achieved. We note that the inherent anisotropy is included in the simulation as the thermal boundary resistance (TBR), which was previously determined for GeTe-Sb_2_Te_3_.^10^ Our previous study^2^ has shown good agreement between simulated results using this modeling approach and the experimental data in terms of expected switching current values. This shows that the simulation framework sufficiently describes the expected device behavior and can also be applied in this study.

**Figure 7:**
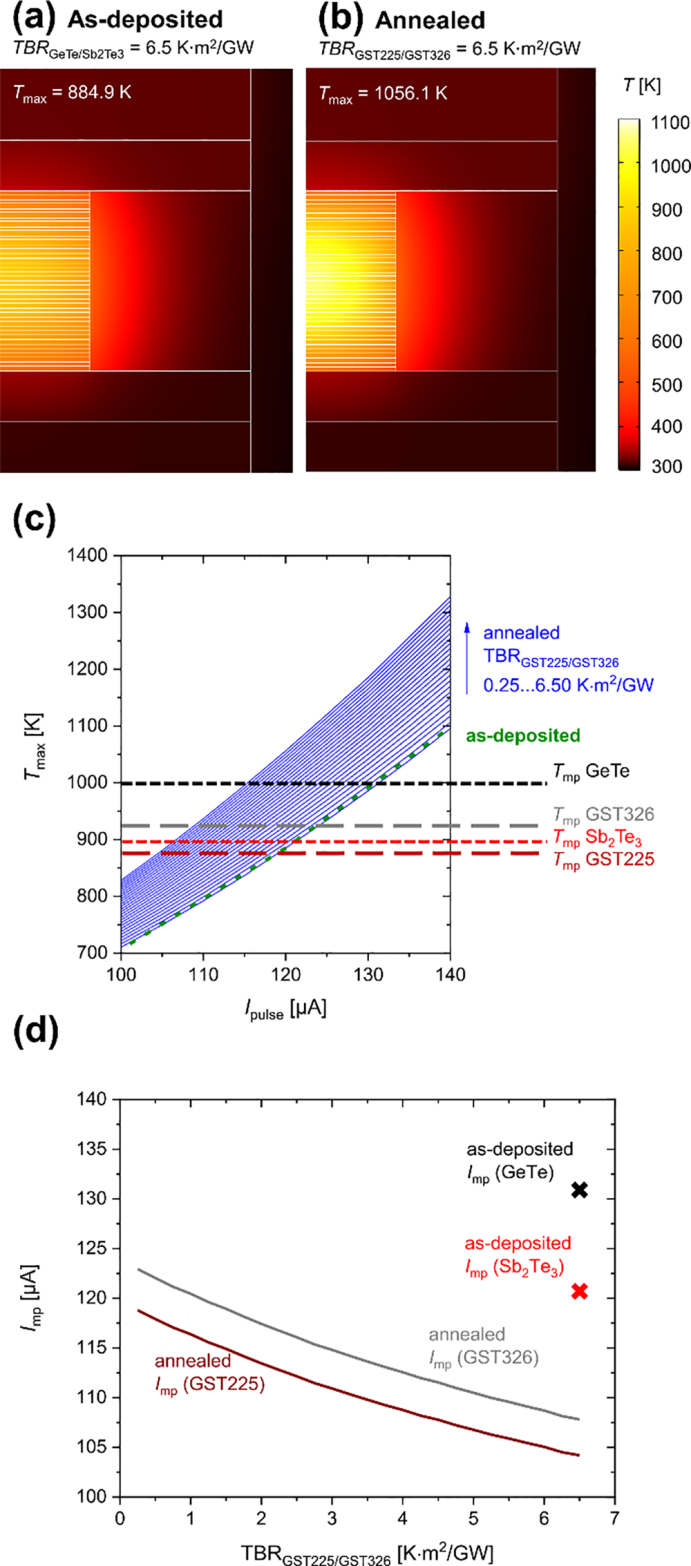
(a, b) Simulated temperature distribution after a RESET pulse of 120 μA and 60 ns in a superlattice GeTe/Sb_2_Te_3_ CSL memory device showing the situation before (GeTe/Sb_2_Te_3_ CSL) and after annealing (Ge_3_Sb_2_Te_6_ and Ge_2_Sb_2_Te_5_ CSL; where Ge_3_Sb_2_Te_6_ abbreviated as GST326 and Ge_2_Sb_2_Te_5_ abbreviated as GST225). (a) as-deposited CSL with TBR_GeTe/Sb_2_Te_3__ = 6.5 K m^2^/GW, and (b) annealed CSL with TBR_GST225/GST326_ = 6.5 K m^2^/GW. Due to unavailability of data in the literature for the CSL after annealing in panel (b), the TBR_GST225/GST326_ of the Ge_2_Sb_2_Te_5_–Ge_3_Sb_2_Te_6_ CSL is shown with the highest assumed value to show the two extrema. The simulated device geometry is shown in Figure S7. (c, d) Simulated CSLs for different TBRs assumed for the annealed CSL. (c) Simulated maximum temperatures depending on the current pulse *I*_pulse_ of the as-deposited case in green (TBR_GeTe/Sb_2_Te_3__ = 6.5 K m^2^/GW) and of the annealed lattice (blue) with various TBR_GST225/GST326_ between 0.25 K m^2^/GW and 6.5 K m^2^/GW in steps of 0.25 K m^2^/GW. The melting temperatures^43−45^ of GeTe and Sb_2_Te_3_ relevant for the as-deposited case and of Ge_2_Sb_2_Te_5_ and Ge_3_Sb_2_Te_6_ for the annealed case are indicated in the graph. (d) Minimum current pulse to reach the melting temperatures of the respective PCMs (*I*_mp_) for the as-deposited CSL and for the annealed CSL depending on the TBR_GST225/GST326_ between 0.25 K m^2^/GW and 6.5 K m^2^/GW. *I*_mp_ was extracted from the data shown in panel (c).

Figure 7 shows the simulated temperature distribution of the CSL device after the RESET pulse (60 ns and 120 μA). The simulation was conducted with the software COMSOL Multiphysics.^42^ The model considers the Seebeck effect, the thermal resistances, including boundaries, and the electric resistances, including boundaries. The details on the parameters entered in the simulation and on the selected simulation modules are included in the Supporting Information. Ideal CSLs with flat interfaces and periodic layer structures were assumed to simplify the simulation design in both cases (before and after annealing). Thus, we selected experimentally determined average layer thicknesses to conduct the simulation. We note that this will provide generalized insight into changes expected on the device level, but real devices may show variability, for example, due to the lamellar structure after annealing at 350 °C (as depicted in Figure 6).

Figures 7a and 7b show the simulated temperature development in the device cross-section after the RESET current pulse for (a) the GeTe/Sb_2_Te_3_ CSL (as-deposited) and (b) the Ge_3_Sb_2_Te_6_ and Ge_2_Sb_2_Te_5_ CSL (annealed), where Ge_3_Sb_2_Te_6_ is abbreviated as GST326 while Ge_2_Sb_2_Te_5_ is abbreviated as GST225. Due to the lack of data about the physical properties of the interfaces between Ge_2_Sb_2_Te_5_ and Ge_3_Sb_2_Te_6_, the annealed CSL was simulated with the same TBR as the GeTe/Sb_2_Te_3_ interface. However, we used the materials parameters of Ge_2_Sb_2_Te_5_ and Ge_3_Sb_2_Te_6_ within the respective layers for the annealed CSL.

A symmetric temperature distribution can be found for both the as-deposited and the annealed devices (Figures 7a and 7b), because of the symmetric device geometry. The highest temperature is reached within the CSL structures. The temperature in the TiN electrodes and the Cu and Al vias does not exceed 50 °C due to the high electrical and thermal conductivities of these materials.^46−51^ This observation is important, as the temperature in the metallization must be guaranteed to be below the melting points of the metals used. In Figure 7a, a maximum temperature of 885 K is reached, and in Figure 7b, a maximum temperature of 1056 K is reached (in the respective superlattices). Both temperatures are above the respective melting temperatures of the involved PCM materials (Figure 7a).^52,53,45^ The higher temperature in Figure 7b can be explained by the influence of the different electrical conductivities (Table S1). For lower electrical conductivities, more energy remains in the material, which leads to the development of higher temperatures.

Figure 7c depicts the simulated maximum temperature after the RESET current pulse, depending on the 60 s current pulse between 100 μA and 140 μA for the simulated device. The green curve shows the maximum temperature developed in the GeTe-Sb_2_Te_3_ SL. The maximum temperatures developed after the RESET current pulse for the simulated Ge_2_Sb_2_Te_5_–Ge_3_Sb_2_Te_6_ SLs are shown in blue, depending on the TBR between 0.25 m^2^·K/GW and 6.5 m^2^·K/GW at the Ge_2_Sb_2_Te_5_–Ge_3_Sb_2_Te_6_ interface. We note that there are currently no literature data available that specify the TBR for the Ge_2_Sb_2_Te_5_–Ge_3_Sb_2_Te_6_ interface. Hence, we simulate a parameter sweep of the TBR at this interface to evaluate the temperature developed in the CSLs, depending on the current pulse and the TBR. Since Ge_2_Sb_2_Te_5_ and Ge_3_Sb_2_Te_6_ are more similar than GeTe and Sb_2_Te_3_, we presume the TBR_GST225/GST326_ to be smaller than 6.5 m^2^·K/GW. All simulated Ge_2_Sb_2_Te_5_–Ge_3_Sb_2_Te_6_-based devices with a TBR_GST225/GST326_ equal to or bigger than 0.5 m^2^·K/GW reach higher temperatures in the superlattice after the RESET current pulse, compared to the as-deposited device (Figure 7c). Additionally, it must be considered that the melting temperatures of Ge_2_Sb_2_Te_5_ and Ge_3_Sb_2_Te_6_ of the annealed materials are below the melting temperature of GeTe and Sb_2_Te_3_.^52,53,45^ This simulated trend is remarkable because it confirms that, also, after annealing or after a high number of thermal events (RESET processes), it is expected that the functionality of the device is maintained, possibly even improved, because it may need a lower reset current for the melt-quench process. This would lead to a better energy efficiency of these iPCM devices upon layer transformation.

Figure 7d shows the required minimum current pulse required to reach the melting point (*I*_mp_) of the respective PCM materials. This graph compares *I*_mp_ for the as-deposited CSL and the annealed CSL depending on the TBR_GST225/GST326_. *I*_mp_ of the respective PCM was extracted by fitting the *I*_pulse_–*T*_max_ graphs, depending on the TBR_GST225/GST326_. It must be considered that the melting temperature of both PCMs included must be reached for device functionality. Since all four CSLs have different melting temperatures, different temperatures had to be reached for the as-deposited and the annealed SL. For the as-deposited GeTe-Sb_2_Te_3_ SL, an *I*_mp_ value of ∼131 μA is required, so that the temperature developed after the current pulse reaches *T*_mp_(GeTe) = 998 K,^43^ as can be seen in Figure 7d. An obvious impact of the CSL type and the TBR at the interface is visible after annealing. To reach the melting temperature of Ge_3_Sb_2_Te_6_ after annealing for TBR_GST225/GST326_ = 6.5 m^2^ K/GW, 17% less current is required than for the as-deposited case (Figure 7d). Even when the actual TBR_GST225/GST326_ is unknown, the trend clearly shows that, for all analyzed values of TBR_GST225/GST326_, the annealed CSL requires lower currents to melt the CSL in the RESET process (Figure 7d). The reasons are the above-mentioned lower melting temperatures of Ge_2_Sb_2_Te_5_ and Ge_3_Sb_2_Te_6_, compared to Sb_2_Te_3_ and GeTe,^52,53,45^ and the lower thermal and electrical conductivities of Ge_2_Sb_2_Te_5_ and Ge_3_Sb_2_Te_6_, which will reduce the heat dissipation in the SL.^10,54,55,2,56^ Although we observe a clear trend in the simulation results, it must be noted that there might be a divergence in the absolute current values when transferring the simulated device into an experiment. This limitation is based on the simplifications in the simulation design, as discussed in the Experimental Section. The impact of the process conditions on the material properties is of significant relevance, as seen in the vast parameter range of electrical and thermal material parameters in the literature. Nevertheless, the simulation result shows promise for stable CSL-based PCM devices with potentially even improved switching properties, which remain to be confirmed in an actual experimental setting in future work.

## 3 Conclusion

In summary, intermixing and ordering phenomena within the GeTe-Sb_2_Te_3_ CSL upon heat treatment are studied using state-of-the-art nanoanalytical methods such as APT and TEM. To the best of our knowledge, this is the first APT study done on GeTe-Sb_2_Te_3_ CSLs heat-treated up to 375 °C.

More precisely, we have shown here that these devices are already highly intermixed in the pristine state, i.e. off-stoichiometric with deviations up to ∼13 at. %, which is usually not discussed in the literature. Moreover, we prove here that the initial GeTe-Sb_2_Te_3_ layers evolve during heat treatment into Ge_2_Sb_2_Te_5_ and Ge_3_Sb_2_Te_6_ lamellae, which remain stable even after 24 h of annealing time at 350 °C. This implies that the Ge_3_Sb_2_Te_6_ phase is stable. We discuss here that the stability of the Ge_3_Sb_2_Te_6_ phase is due to the preservation of the vdW-like gaps within the PCM layer.

By FEM simulations of CSL-based memory elements, we compared the temperature development and required RESET current pulses for the initial GeTe-Sb_2_Te_3_ and annealed (Ge_2_Sb_2_Te_5_–Ge_3_Sb_2_Te_6_) superlattices. Our results suggest that the electrothermal properties of the CSL are not negatively impacted and that the formation of stacked layers of Ge_2_Sb_2_Te_5_–Ge_3_Sb_2_Te_6_ blocks could even lead to an improved device performance (i.e., a reduction in the RESET current).

Hence, this study proves the feasibility to quantitatively determine the chemical intermixing and ordering taking place within the CSL thin film. These findings can be extrapolated to the behavior of CSLs during the switching phenomenon. This switching-induced intermixing can be quantified for a large class of phase-change-based devices, such as iPCM and PCM, which is crucial for a better understanding of the underlying device functioning and failure mechanisms.

## 4 Experimental Section

### 4.1 Sample Fabrication

GeTe- and Sb_2_Te_3_-based CSLs with 20 repetitions have been sputter-deposited from stoichiometric targets of 4N purity on a Si(111) substrate with ρ > 5 mΩ cm at a deposition temperature of 200 °C. Superficial oxide on the substrate was removed beforehand by a wet etch in aqueous hydrofluoric acid.

The DC power was 35 W with an argon gas flux set to 35 sccm. The growth rate of GeTe and Sb_2_Te_3_ is 0.1 nm s^–1^. The deposition was carried out at a base pressure of *p* = 10^–8^ mbar in an HV sputtering system with sputtering sources in confocal geometry and individually controlled shutters that allow for precise control of the serial deposition. Moreover, the CSL is capped by an insulating sputter-deposited capping layer of 20 nm (ZnS)_80_(SiO_2_)_20_ to prevent the CSL from oxidation during the subsequent post-annealing procedure. The same parameters as the CSL layer have been used to deposit the (ZnS)_80_(SiO_2_)_20_ layer by magnetron sputtering.

The CSL samples were then postannealed in a tube furnace in an Ar atmosphere with a 150 sccm Ar flow and a ramp of 5 K min^–1^ to temperatures of 250, 275, 300, 325, 350, and 375 °C at which they were kept for 30 min. Additionally, one CSL sample was post-annealed to 350 °C for 24 h.

### 4.2 Sample Characterization

For overall structural analysis of the thin film, superlattices were done by using X-ray diffraction (XRD) with the Bruker D8 Discover setup equipped with a Goebel mirror, a Ge(220) asymmetric channel-cut monochromator, and a Eulerian cradle.

The structural analysis down to the atomic level was performed by high-resolution (scanning) transmission electron microscopy (HR STEM) with an FEI Titan ChemiSTEM 80–300 Cs-STEM and equipped with an imaging spherical aberration corrector on TEM lamellae and APT tips in the case of 350 °C 0.5 h annealed CSL and as deposited CSL, respectively. Acceleration voltages of 300 kV for the HR (S)TEM images as well as 200 kV for annular dark field (ADF) and high angle annular dark field (HAADF) STEM (Z-contrast) images were used. The images were acquired in the [11̅0] zone of Sb_2_Te_3_ or the stable GST (or, in some cases, out of the zone, but with *c* -axis in the image plane).

The composition intermixing was studied at the nanoscale using APT. The investigations were carried out with the CAMECA LEAP 4000X Si local electrode atom probe with laser pulses of 355 nm wavelength (UV), 10 ps laser pulse duration, and 17 pJ - 22 pJ pulse energy in ultrahigh vacuum (10^–10^ mbar). The pulse rate was set to 200–250 kHz and the specimen base temperature was kept at *T* = 40 K. We mention here that, for each CSL sample, at least three successful atom probe measurements were conducted to ensure reproducibility.

Samples for TEM as well as for the atom probe tomography (APT) were prepared with the Dual-Beam Helios NanoLab 650 System and finally polished at low energy (500 eV) to remove residual FIB-induced amorphization.^57^ For the APT investigations, needle-shaped specimens were prepared using FIB milling at the Combined SEM/FIB Dual-Beam Helios NanoLab 650 System, as described in ref (58). To minimize beam-induced damage, a low energy (5 keV) Ga^+^ beam was used at the final ion-milling stage as described in ref (57).

### 4.3 Device Simulation

The electrothermal device simulations were conducted with the software COMSOL Multiphysics.^42^ For this purpose, the modules “Heat Transfer in Solids” and “Electric Currents” were employed. Thus, the model considers several relevant electrical and thermal parameters such as the Seebeck effect, the thermal resistances, including boundaries, and the electric resistances, including boundaries. In Figure S7, the geometries of the simulated devices including dimensions are schematically visualized. Figure S7b shows where which boundary condition was implemented in the model. An overview of the parameters entered into the simulation is available in Table S1. Other material parameters, which were already included in the COMSOL material database, are not shown in this table.

After the simulation design was set up and the input values and boundary conditions were added, the model was distributed into a physics-controlled mesh for the simulation. A time-dependent simulation was selected to store the data of different times during the current pulse (60 ns) between 100 and 140 μA. A current pulse, representing the reset pulse, was selected according to ref (2). The current pulse was applied between copper and aluminum electrodes, and the temperature development in the superlattice was simulated. The data were taken directly after the reset pulse to show the maximum temperature developed in the device.

## Supporting Information Available

The Supporting Information is available free of charge at··· The Supporting Information includes: The Supporting Information is available free of charge at https://pubs.acs.org/doi/10.1021/acsnano.4c13450.

(a) Supplementary XRD results used for the determination of the CSL superlattice peak and the analysis of the (00l) family of 350 °C annealed Sb_2_Te_3_–GeTe superlattice to determine the *c*-lattice constant of Ge_2_Sb_2_Te_5_; (b) STEM investigation of a needle-shaped APT tip showing the Sb_2_Te_3_–GeTe superlattices after deposition and annealing at 350 °C (their corresponding crystal structures, as well as the tuples present, are provided); (c) supplementary APT investigations further highlighting the intermixing behavior after deposition and annealing and the presence of various phases with their respective stoichiometries; (d) parameters employed for the device simulation (PDF)
