# Supplementary material for: Thermally Assisted Atomic-Scale Intermixing and Ordering in GeTe–Sb2Te3 Superlattices
Source: ACS Nano. 2025 Feb 6;19(6):6130–41. doi: 10.1021/acsnano.4c13450 (PMC11841033; doi:10.1021/acsnano.4c13450)
Supplement: Supplementary file 1 — nn4c13450_si_001.pdf [file nn4c13450_si_001.pdf]

Supporting Information

Thermally assisted atomic-scale intermixing and ordering in GeTe-Sb_2Te_3 superlattices

Oana Cojocaru-Mirédin[1,2,] Jasmin-Clara Bürger[3], Nikita Polin [1,4], Alexander Meledin[5], Joachim Mayer[5], Matthias Wuttig [1,6,7], Alwin Daus[3]*

[1] I. Institute of Physics (IA), RWTH Aachen University, 52056 Aachen, Germany

[2] INATECH, University of Freiburg, Emmy-Noether-Straße 2, 79110 Freiburg, Germany

[3] IMTEK, University of Freiburg, Georges-Köhler-Allee 103, 79110 Freiburg, Germany

[4] Max-Planck-Institut für Eisenforschung GmbH, 40237 Düsseldorf, Germany

[5] Ernst Ruska-Centre (ER-C-2), Forschungszentrum Jülich, 52428 Jülich, Germany

[6] Jülich-Aachen Research Alliance (JARA-HPC and JARA-FIT), RWTH Aachen University, 52056 Aachen, Germany

[7] Peter-Grünberg-Institute (PGI 10), Forschungszentrum Jülich, 52428 Jülich, Germany

[*]Corresponding author: oana.cojocaru-miredin@inatech.uni-freiburg.de

1 Results

CSL Bulk Structure evolution down to the nanoscale

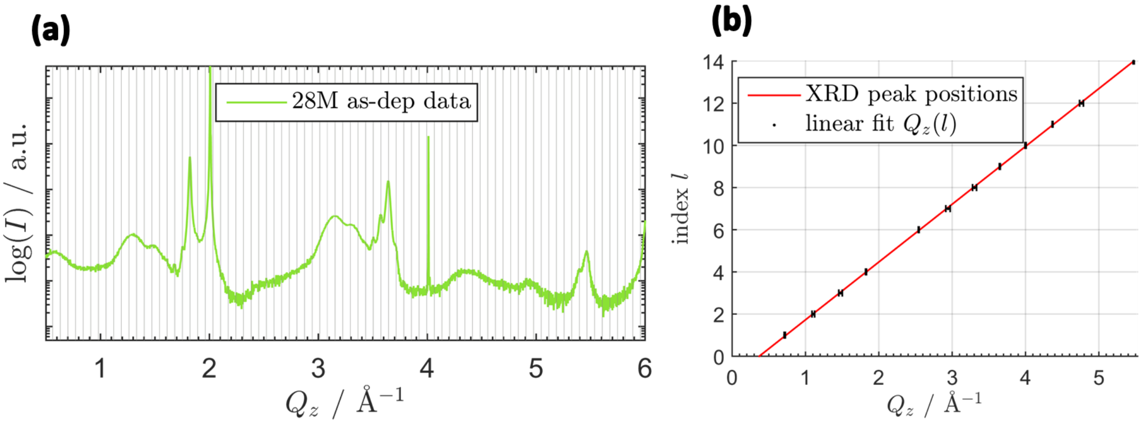

Fig. S1. XRD analysis of ~330 nm as-deposited and annealed Sb_2Te_3 –GeTe superlattices sputter deposited on Si(111) substrate. (a) *T2T symmetrical XRD scan of the as-deposited CSL film where the lattice determined for the supercell from the fit is drawn in grey.* ***(b)*** *Analysis of the (00l) family of 350 °C annealed Sb_2Te_3 –GeTe superlattice to determine the c lattice constant of $Ge_2Sb_2Te_5$.*

Fig. S2(a) shows the scanning transmission electron microscopy (STEM) image of a needle-shaped atom probe tomography (APT) tip prepared by the focused ion beam (FIB) procedure with an intensity profile extracted from the rectangular region as indicated in

the image. The chemical identification of the layers is possible given that the contrast in STEM images is roughly proportional to $Z^{1.7}$, where Z is the atomic number[1],[2], The Sb_2Te_3 layers appear brighter as the GeTe layer ($Z_{Sb}>Z_{Ge}$, with $Z_{Sb}=51$ and $Z_{Ge}=32$). Moreover, **Fig. S2(b)** shows the as-deposited chalcogenide superlattice (CSL) at a higher magnification displaying distinctive crystal structures of Sb_2Te_3 and GeTe separated by the 'vdW-like' gaps. A wide variation of tuples was identified in **Fig. S3**, namely 7-, 9- and 11-tuples.

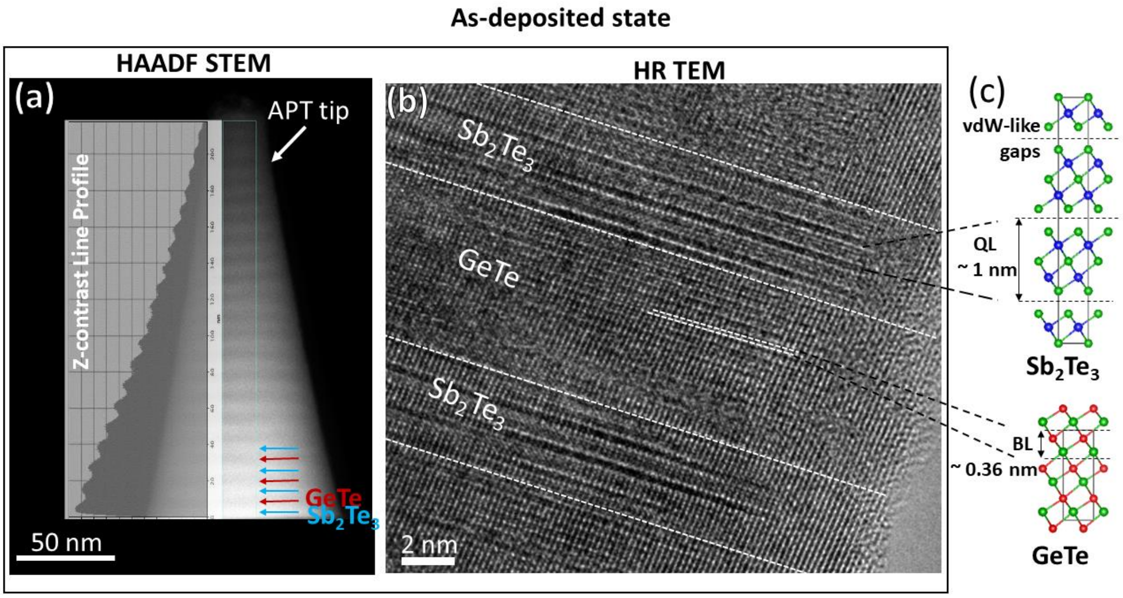

Fig. S2. TEM and STEM investigations of ~220 nm as-deposited Sb_2Te_3–GeTe superlattices together with corresponding crystal structures. *(a1) HAADF STEM and (a2) High Resolution (HR) TEM micrographs of APT needle-shaped specimen of the CSL layer in the as-deposited state. Additionally, corresponding crystal structures for GeTe and Sb_2Te_3 are displayed. Sb_2Te_3 regions can be clearly distinguished from GeTe thanks to the 'vdW-like' gaps.*

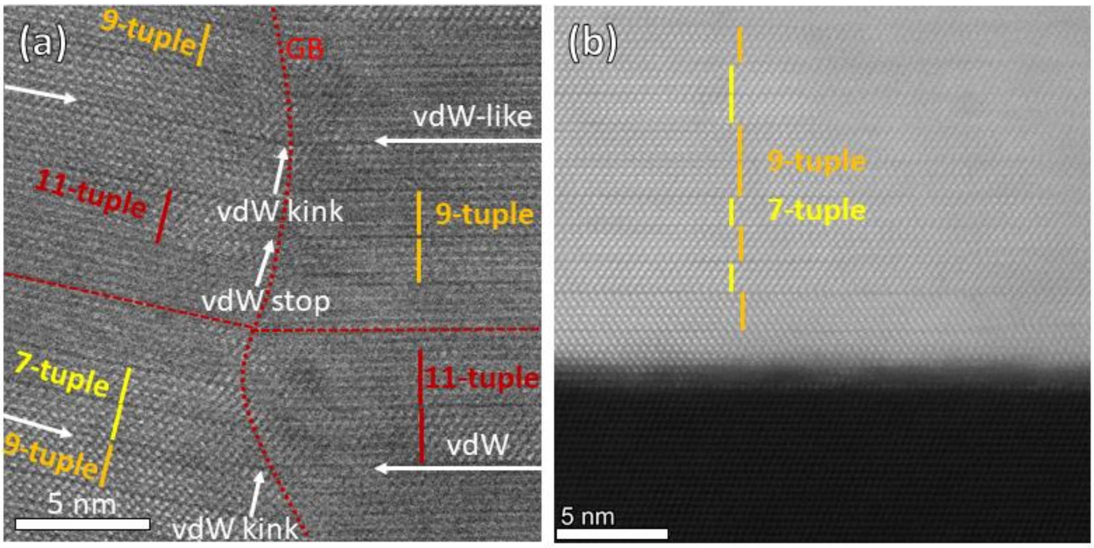

__Fig. S1. HAADF STEM analysis of 350°C annealed samples at (a) high and (b) medium magnification.__ *In (a) the grain boundary region is enlarged showing the presence of 7-, 9- and 11-Tuples. The presence of 7-tuple is confirmed again in region shown in (b), which is a region close to the bottom of the CSL layer close to the Si(111) substrate.*

2 Intermixing in CSL upon annealing

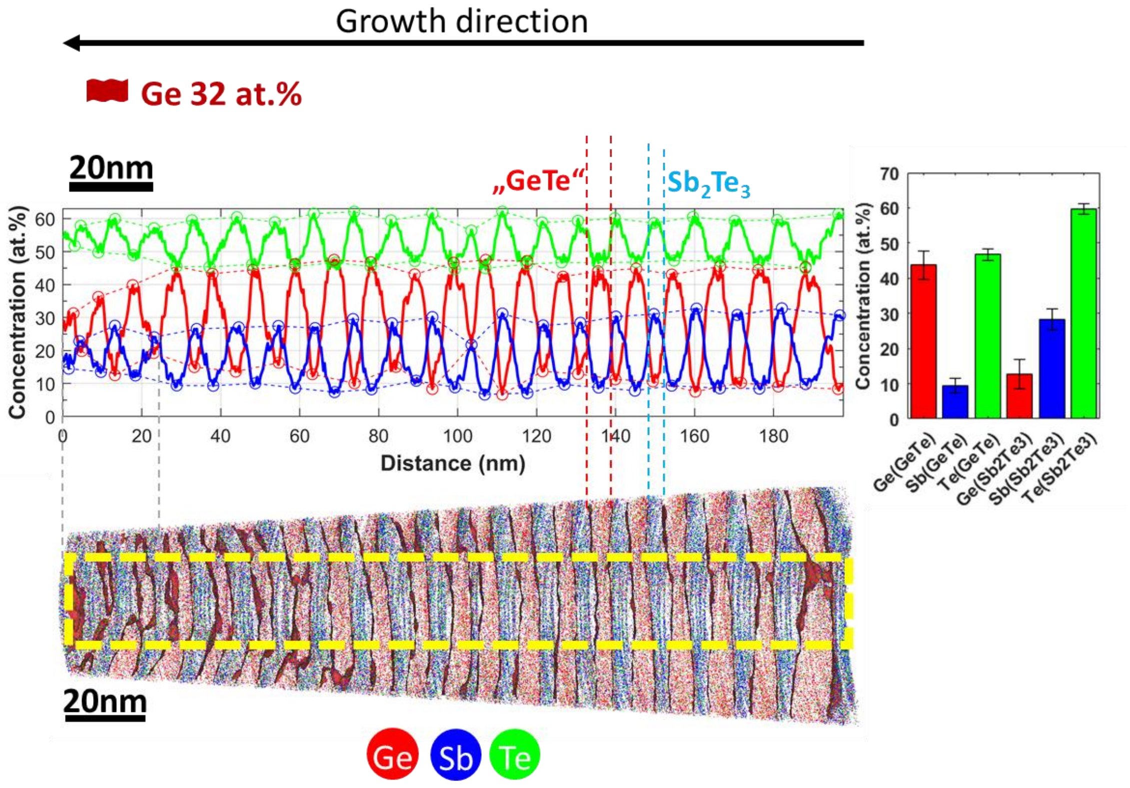

Fig. S2. *The statistical determination of the composition inside the layers using APT 1D concentration profiles.*

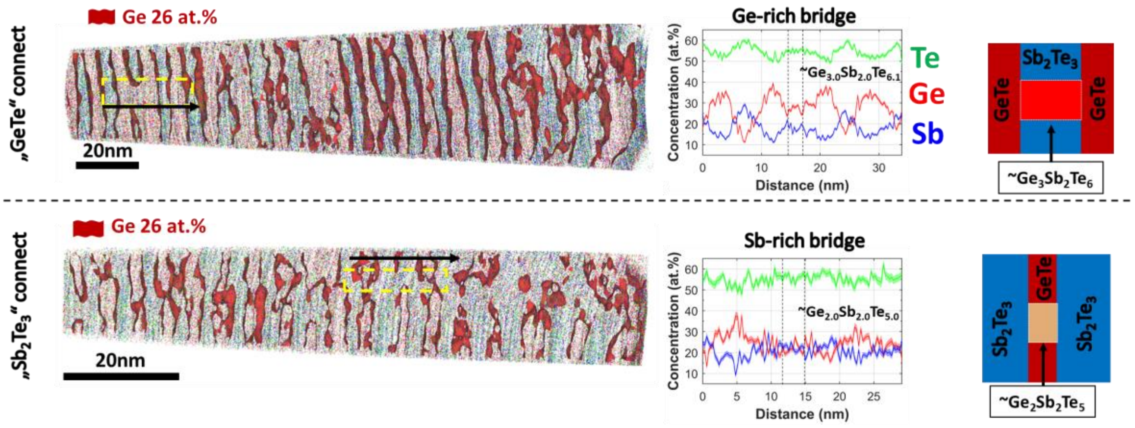

Fig. S5. *APT investigation of 275°C annealed CSL showing regions where neighbouring layers are connected (yellow dashed rectangles) called "bridges". Left: 3D cropped elemental map; middle: 1D concentration profiles; right: schematic representation of the intermixing inside the bridges.*

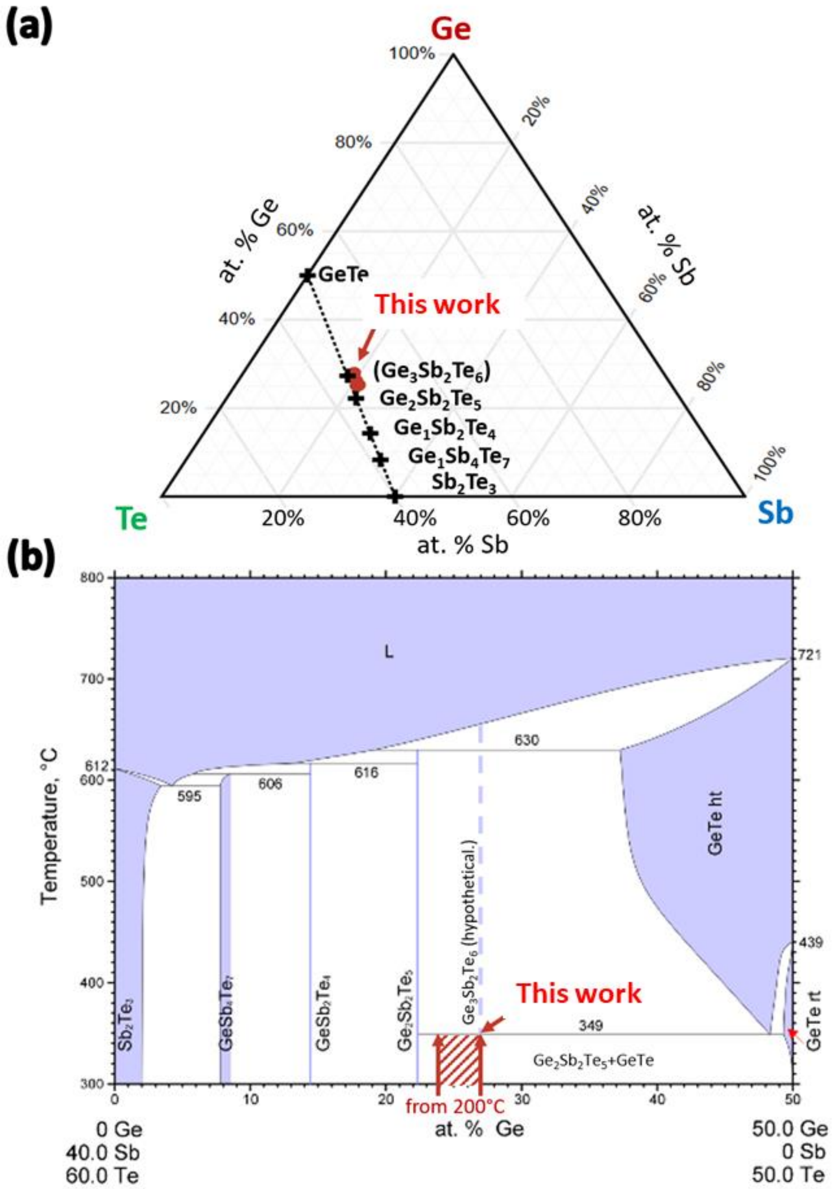

Fig. S6. *Ternary and pseudo-binary GeTe-Sb_2Te_3 phase diagram with the specimen compositions investigated within this work. The binary phase diagram was substracted from the work of Bordas et al.*[3]

3. Device simulation

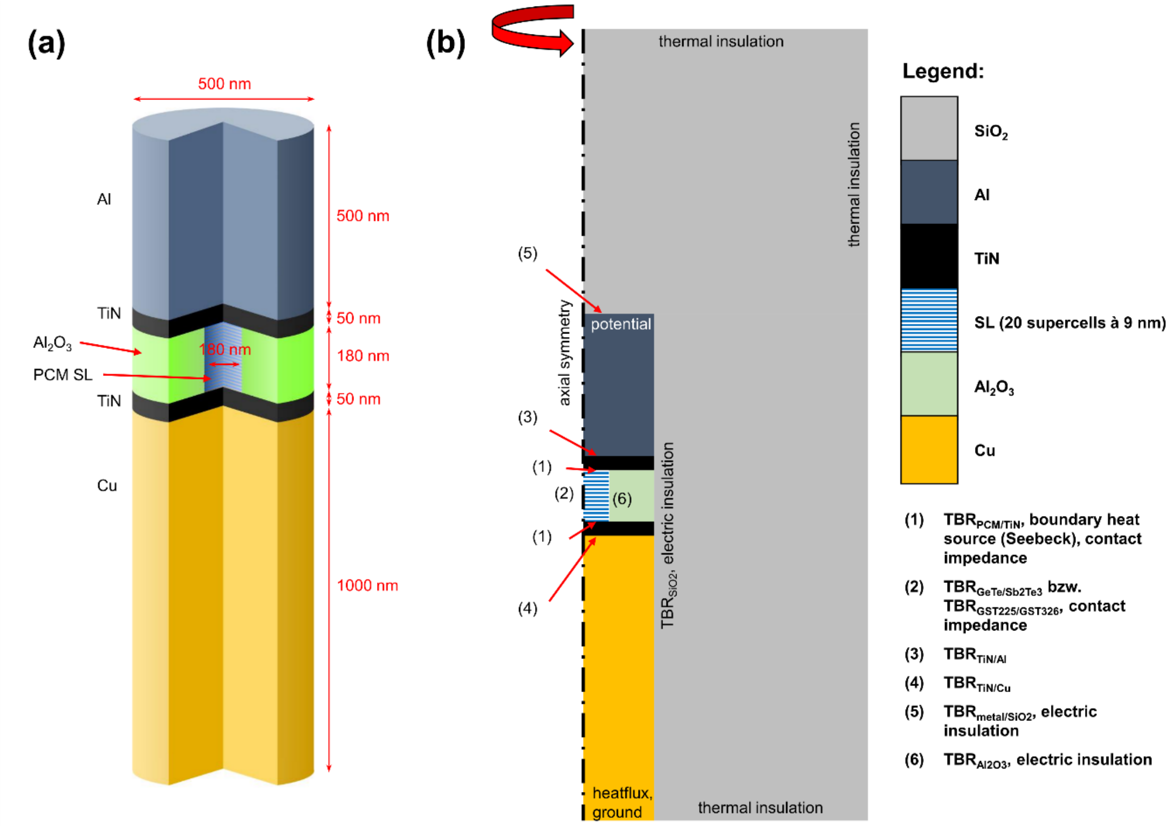

Fig. S7: *Schematic design of the simulation: (a) 3D visualization of the simulated device. The* SiO_2 *bulk material is not shown in (a). (b) Schematic section (half cross-section) of the simulated phase change memory (PCM) device with indicated boundary conditions considered in the COMSOL*[4] *simulation. The geometries and materials are selected based on the experimental results shown in Fig. 2 and based on the literature on comparable CMOS-integrated devices.*

Table S1: Material parameters entered in the simulation. Material parameters already included in COMSOL are not listed in this table.

**Physical quantity**	Material	Value	Notes and References
**Electrical conductivities**	SiO_2	$1 \cdot 10^{-12}$ S/m	[5], [6]
	TiN	$2 \cdot 10^{4}$ S/cm	[7],[8],[9]
	Al_2O_3	$1 \cdot 10^{-12}$ S/m	[5]
	Cu	$5.8 \cdot 10^{7}$ S/m	[4]

	$Ge_2Sb_2Te_5$	Temperature-dependent between $3.4 \cdot 10^3$ S/cm - $7 \cdot 10^3$ S/cm	[10],[5]
	$Ge_3Sb_2Te_6$	$2 \cdot 10^3$ S/cm	[11]
	Sb_2Te_3	$5 \cdot 10^3$ S/cm	[5]
	GeTe	$5 \cdot 10^3$ S/cm	[5]
**Thermal conductivi-ties**	GeTe	2 W/(m·K)	[12]
	Sb_2Te_3	3 W/(m·K)	[13],[14]
	$Ge_2Sb_2Te_5$	Temperature-dependent between 0.18 W/(m·K) and 1.25 W/(m·K)	[15]
	$Ge_3Sb_2Te_6$	0.82 W/(m·K)	[12]
	SiO_2	1.3 W/(m·K)	[16]
**Density**	SiO_2	2200 kg/m^3	[17]
	TiN	522 kg/m^3	[18]
	Al_2O_3	3965 kg/m^3	[19]
	$Ge_2Sb_2Te_5$	6 g/cm^3	[20]
	$Ge_3Sb_2Te_6$	6.0 g/cm^3	[21]
	Sb_2Te_3	6500 kg/m^3	[5]
	GeTe	6140 kg/m^3	[5]
**Heat capa-city *C***	SiO_2	730 J/(kg·K)	[22]
	TiN	50 J/(mol·K)	[23]
	Al_2O_3	C_{SiO2}	Assumption as SiO_2
	$Ge_2Sb_2Te_5$	212 J/(kg·K)	[24]
	$Ge_3Sb_2Te_6$	24 J/(kg·K)	[12]
	Sb_2Te_3	157 J/(kg·K)	[5]
	GeTe	134 J/(kg·K)	[5]
**Thermal Boundary**	TBR_{TiN}	56 m^2·K/GW	[5]
	$TBR_{TiN/Al}$	1.5 m^2·K/GW	[25]
	$TBR_{TiN/Cu}$	$TBR_{TiN/Al}$	Assumption as $TBR_{TiN/Al}$
	$TBR_{SbTe/GeTe}$	6.5 m^2·K/GW	[5]

**Resistance TBR**	TBR_{SiO2}	50 $m^2 \cdot K/GW$	[5],[26],[27]
	TBR_{Al2O3}	50 $m^2 \cdot K/GW$	[5]
**Seebeck coefficient *S***	S_{GST225}	150 uV/K	[28],[5]
	S_{GST326}	150 uV/K	[28]
	S_{SbTe}	150 uV/K	[29]
	S_{GeTe}	150 uV/K	[30]
	S_{TiN}	-4 … -50 µV/K	[31]
**Relative Permittivity**	$Ge_2Sb_2Te_5$	16	[32]
	$Ge_3Sb_2Te_6$	12.1	[32]
	Sb_2Te_3	16.5	[5]
	GeTe	16.5	[5]
**Contact impedance**	PCM-metal	$5.75 \cdot 10^{-6}\ \Omega \cdot cm^2$	[5]
	GeTe/Sb_2Te_3	$2.5 \cdot 10^{-6}\ \Omega \cdot cm^2$	[5]

For the $Ge_2Sb_2Te_5$-$Ge_3Sb_2Te_6$-based simulation model, we were missing data for the TBR between the GST layers in the annealed SL. Due to the ternary combination of the same elements, the GST layers are more similar than the GeTe and the Sb_2Te_3 layers. Hence, the $TBR_{GST225/GST326}$ is expected to be smaller than the $TBR_{GeTe/Sb2Te3}$. Therefore, we assumed that the values must be between 0.25 $m^2 \cdot K/GW$ and 6.5 $m^2 \cdot K/GW$. Mainly, the TBR is only given for interfaces between different materials, and 0.25 $m^2 \cdot K/GW$ is already a small value. Ref.[33] gives a value of 1.55 $m^2 \cdot K/GW$ for a carbon nanotube (CNT)-CNT interface. Based on this, we suspect the $TBR_{GST225/GST326}$ may be larger. A constant TBR of 56 $m^2 \cdot K/GW$ was chosen for the PCM-TiN interfaces for all four PCM materials[34],[5],[35]. Thus, the influence of the TBR at the PCM-TiN interface is significantly higher than the TBR at the Al-TiN interface[25],[5]: 1-2 $m^2 \cdot K/GW$. Because we did not find any information about the Cu-TiN interface, we assumed that the TBR of

this interface is of a similar order of magnitude as the TBR at the Al-TiN interface. For this reason, a constant $TBR_{metal/TiN}$ was chosen.

With the simulation, we compare the temperature rise for a CSL device before/after annealing when subjected to Joule heating with a current pulse. The important parameter is the current required to reach the melting temperatures of the PCMs which depends on the different PCM compositions and configuration. In Fig. S7 the simulation design is schematically visualized. In Fig. S7(a) the selected device dimensions are included. Fig. S7(b) shows where which boundary condition was implemented in the model. The most relevant boundary conditions were considered to represent a realistic situation: axial symmetry, thermal insulation, TBR, heat flux, current conservation, electric insulation, contact impedance, and the direction and insertion of the current pulse. The materials were selected to represent a configuration that could be realistic for integration in a CMOS device. In such devices, the memory elements are positioned in one of the top layers[36], [37]. The CSL is typically sputtered layer by layer[5]. The as-deposited GeTe-Sb_2Te_3 CSL represents our experimental design. Hence, the layer thicknesses of 3.8 nm for the GeTe layers and 5.2 nm for the Sb_2Te_3 layers were selected. As vias and metallizations, Al and Cu are typical materials in CMOS chips[38],[39]. TiN, a commonly used material for electrodes in CSL-based memory elements, was chosen for the contacts[5],[36],[37]. The details of the geometrical design of the simulated device are included in Fig. S7(a). As a simplification, a perfect $Ge_2Sb_2Te_5$-$Ge_3Sb_2Te_6$ superlattice with 4 nm $Ge_3Sb_2Te_6$ and 5 nm $Ge_2Sb_2Te_5$ was assumed for the annealed case. Apart from the GeTe-Sb_2Te_3 and $Ge_2Sb_2Te_5$-$Ge_3Sb_2Te_6$ layers, both simulated devices are identical in the geometrical design and the included parameters (Fig. S7). This simulation design allows for comparability of both cells: The simulation of the GeTe-Sb_2Te_3-based device (as-deposited) and the $Ge_2Sb_2Te_5$-$Ge_3Sb_2Te_6$-based device (after annealing). The material properties were

assigned to the individual simulation domains in the simulation model, as shown in Fig. S7. An overview of the entered parameters is added to Table S1. Material parameters, which were already included in the COMSOL® material database are not shown in this table.

For improvement of the computation time and similarly for maintenance of the information value, simplifications were made: In Fig. 2, the formation of 7-tuples, 9-tuples, and 11-tuples within the initial GeTe and Sb_2Te_3 layers was observed. This observation is consistent with the literature for PCMs. Ref.[40] shows clearly the formation and order of the different tuples depending on the GeTe and Sb_2Te_3 layer thicknesses of the as-deposited devices. Experimentally, the effective TBR in a CSL stack is always measured from a superposition of the TBRs at the vdW-like interfaces within a material ("intramaterial interfaces", e.g. at a GeTe-GeTe interface at a vdW-like gap in GeTe) and between the different material layers ("intermaterial interfaces", e.g., at the GeTe-TiN or $Ge_2Sb_2Te_5$-$Ge_3Sb_2Te_6$ interface). Based on prior investigations (Fig. 2 in ref.[15]), we know that the TBR at intramaterial vdW-like gaps must be much smaller than the TBR at intermaterial vdW-like gaps. Thus, we neglected the TBRs at the intramaterial vdW-like gaps in our simulation.

In addition, the specific contact impedance strongly impacts the temperature development in the SL. Because the simulated as-deposited device and the simulated annealed device only differ in the CSL (materials and layer thickness), the contact impedance was only considered at the PCM interfaces within the SLs and the interfaces to the two TiN electrodes. Due to missing information about the specific contact impedance at the $Ge_2Sb_2Te_5$-TiN and $Ge_3Sb_2Te_6$-TiN interfaces in the literature, the same value of $5.75 \cdot 10^{-6}$ $\Omega \cdot cm^2$ as for the GeTe-TiN and Sb_2Te_3-TiN interfaces was chosen according to ref.[5]. For the Sb_2Te_3-GeTe interfaces a specific contact impedance of $2.5 \cdot 10^{-6}$ $\Omega \cdot cm^2$ was

selected based on ref. [5]. For the $Ge_2Sb_2Te_5$-$Ge_3Sb_2Te_6$ interfaces the same contact impedance was assumed.

Above that, the temperature developed in the device depends on the thermal and electrical conductivities. Various values for these physical quantities were found in the literature. The reason is the influence on the material quality by, among others, the synthesis and annealing processes. These processes have a significant impact on the crystallinity of the material and, hence, the material properties[41]. The values for the electrical and thermal conductivities were mainly selected based on previous measurements of samples, which we fabricated with the same equipment and recipes in earlier studies to be consistent with the experimental results in this publication[42],[11],[16],[15],[5]. The values are listed in Table S1. It has to be considered that all entered values strongly depend on the crystallinity[43]. This leads to deviations in the values found in the literature. Thus, the phase transformation in the real CSL-based memory element will also influence the developed temperature due to the set or reset pulses in the real device. This might also lead to differences between the simulation and a similar experimental setup in the device behavior and the actually developed temperature.

Bibliography:

[1] O. Cojocaru-Mirédin, H. Hollermann, A. M. Mio, A. Y. T. Wang, and M. Wuttig, "Role of grain boundaries in Ge-Sb-Te based chalcogenide superlattices," *J. Phys. Condens. Matter*, vol. 31, no. 20, 2019, doi: 10.1088/1361-648X/ab078b.

[2] B. J. Kooi and J. Momand, "High Resolution Imaging of Chalcogenide Superlattices for Data Storage Applications: Progress and Prospects," *Phys. Status Solidi - Rapid Res. Lett.*, vol. 13, no. 4, pp. 1–13, 2019, doi: 10.1002/pssr.201800562.

[3] S. Bordas, M. T. Clavaguer-Mora, B. Legendre, and C. Hancheng, "Phase diagram of the ternary system Ge-Sb-Te: II. The subternary Ge-GeTe-Sb2Te3-Sb," *Thermochim. Acta*, vol. 107, pp. 239–265, 1986, doi: https://doi.org/10.1016/0040-6031(86)85051-1.

[4] "COMSOL Multiphysics v. 5.4." COMSOL AB, Stockholm, Sweden, Sweden. [Online]. Available: www.comsol.com.

[5] A. I. Khan *et al.*, "Ultralow-switching current density multilevel phase-change memory on a flexible substrate.," *Science*, vol. 373, no. 6560, pp. 1243–1247, Sep. 2021, doi: 10.1126/science.abj1261.

[6] M. M. Gauthier, "Engineered Materials Handbook Desk Edition." ASM International, Nov. 01, 1995. doi: 10.31399/asm.hb.emde.9781627082006.

[7] P. Patsalas and S. Logothetidis, "Optical, electronic, and transport properties of nanocrystalline titanium nitride thin films," *J. Appl. Phys.*, vol. 90, no. 9, pp. 4725–4734, Nov. 2001, doi: 10.1063/1.1403677.

[8] L. Assaud, K. Pitzschel, M. Hanbücken, and L. Santinacci, “Highly-Conformal TiN Thin Films Grown by Thermal and Plasma-Enhanced Atomic Layer Deposition,” *ECS J. Solid State Sci. Technol.*, vol. 3, no. 7, p. P253, 2014, doi: 10.1149/2.0141407jss.

[9] P. Patsalas, C. Charitidis, S. Logothetidis, C. A. Dimitriadis, and O. Valassiades, “Combined electrical and mechanical properties of titanium nitride thin films as metallization materials,” *J. Appl. Phys.*, vol. 86, no. 9, pp. 5296–5298, Nov. 1999, doi: 10.1063/1.371514.

[10] L. Adnane *et al.*, “High temperature electrical resistivity and Seebeck coefficient of Ge2Sb2Te5 thin films,” *J. Appl. Phys.*, vol. 122, no. 12, p. 125104, Sep. 2017, doi: 10.1063/1.4996218.

[11] E.-R. Sittner, K. S. Siegert, P. Jost, C. Schlockermann, F. R. L. Lange, and M. Wuttig, “(GeTe)x–(Sb2Te3)1–x phase-change thin films as potential thermoelectric materials,” *Phys. status solidi*, vol. 210, no. 1, pp. 147–152, Jan. 2013, doi: https://doi.org/10.1002/pssa.201228397.

[12] K. S. Siegert, “Thermal Properties of Phase-Change Materials from lattice dynamics to thermoelectricity,” RWTH Aachen University, 2015.

[13] J.-E. Hong, S.-K. Lee, and S.-G. Yoon, “Enhanced thermoelectric properties of thermal treated Sb2Te3 thin films,” *J. Alloys Compd.*, vol. 583, pp. 111–115, 2014, doi: https://doi.org/10.1016/j.jallcom.2013.08.164.

[14] F. Rieger *et al.*, “Low intrinsic c-axis thermal conductivity in PVD grown epitaxial Sb2Te3 films,” *J. Appl. Phys.*, vol. 123, no. 17, p. 175108, May 2018, doi: 10.1063/1.5025491.

[15] H. Kwon, A. I. Khan, C. Perez, M. Asheghi, E. Pop, and K. E. Goodson, “Uncovering Thermal and Electrical Properties of Sb2Te3/GeTe Superlattice Films,” *Nano Lett.*, vol. 21, no. 14, pp. 5984–5990, Jul. 2021, doi: 10.1021/acs.nanolett.1c00947.

[16] I. Chen, “A compact thermal model for segmented nanowire phase-change memory cells,” Standford University, 2008.

[17] E. Bassous, “Fabrication of novel three-dimensional microstructures by anisotropic etching of (100) and (110) Silicon,” *IEEE Trans. Electron Devices*, vol. 25, no. 10, p. 1178, 1978.

[18] A. Hashim and Z. Hamad, “Developments in Polymer Nanocomposites for Modern Biomedical and Industrial Applications: A Review,” *Res. J. Agric. Biol. Sci.*, vol. 14, pp. 1–9, Aug. 2019, doi: 10.22587/rjabs.2019.14.2.1.

[19] M. Aqib, A. Hussain, H. M. Ali, A. Naseer, and F. Jamil, “Experimental case studies of the effect of Al2O3 and MWCNTs nanoparticles on heating and cooling of PCM,” *Case Stud. Therm. Eng.*, vol. 22, p. 100753, 2020, doi: https://doi.org/10.1016/j.csite.2020.100753.

[20] I.-D. Simandan *et al.*, “Influence of Deposition Method on the Structural and Optical Properties of Ge2Sb2Te5,” *Materials (Basel).*, vol. 14, p. 3663, Jun. 2021, doi: 10.3390/ma14133663.

[21] A. Jain *et al.*, “Commentary: The Materials Project: A materials genome approach to accelerating materials innovation,” *APL Mater.*, vol. 1, no. 1, p. 11002, Jul. 2013, doi: 10.1063/1.4812323.

[22] Az. U. Limited, “AZo MATERIALS, AZoNetwork UK Limited, Manchester,” 2024. https://www.azom.com/properties.aspx?ArticleID=1114, access 27/2/2024

[23] M. W. Barsoum *et al.*, “Thermal properties of Ti4AlN3,” *J. Appl. Phys.*, vol. 87, no. 12, pp. 8407–8414, Jun. 2000, doi: 10.1063/1.373555.

[24] D. Tripathi and R. Hegde, “Optimally designed tunable phase change material-based narrowband perfect absorber,” *J. Nanophotonics*, vol. 17, no. 1, p. 16004, Jan. 2023, doi: 10.1117/1.JNP.17.016004.

[25] E. Bozorg-Grayeli, J. P. Reifenberg, M. A. Panzer, J. A. Rowlette, and K. E. Goodson, “Temperature-Dependent Thermal Properties of Phase-Change Memory Electrode Materials,” *IEEE Electron Device Lett.*, vol. 32, no. 9, pp. 1281–1283, 2011, doi: 10.1109/LED.2011.2158796.

[26] S. Durai, S. Raj, and A. Manivannan, “Impact of Thermal Boundary Resistance on the Performance and Scaling of Phase-Change Memory Device,” *IEEE Trans. Comput. Des. Integr. Circuits Syst.*, vol. 39, no. 9, pp. 1834–1840, 2020, doi: 10.1109/TCAD.2019.2927502.

[27] S. Kumari, S. Durai, and A. Manivannan, “Impact of process-induced variability on multi-bit phase change memory devices,” *Microelectronics J.*, vol. 130, p. 105638, 2022, doi: https://doi.org/10.1016/j.mejo.2022.105638.

[28] W. Ibarra-Hernández and J.-Y. Raty, “Ab initio density functional theory study of the electronic, dynamic, and thermoelectric properties of the crystalline pseudobinary chalcogenide ${\text{(GeTe)}}_{x}/({\mathrm{Sb}}_{2}{\mathrm{Te}}_{3})\phantom{\rule{0.28em}{0ex}}(x=1,\phantom{\rule{0.28e,” *Phys. Rev. B*, vol. 97, no. 24, p. 245205, Jun. 2018, doi: 10.1103/PhysRevB.97.245205.

[29] R. Venkatasubramanian, T. Colpitts, E. Watko, M. Lamvik, and N. El-Masry, “MOCVD of Bi2Te3, Sb2Te3 and their superlattice structures for thin-film thermoelectric applications,” *J. Cryst. Growth*, vol. 170, no. 1, pp. 817–821, 1997, doi: https://doi.org/10.1016/S0022-0248(96)00656-2.

[30] J. Li, Z. Chen, X. Zhang, Y. Sun, J. Yang, and Y. Pei, “Electronic origin of the high thermoelectric

performance of GeTe among the p-type group IV monotellurides," *NPG Asia Mater.*, vol. 9, no. 3, pp. e353–e353, 2017, doi: 10.1038/am.2017.8.

[31] A. Faraclas *et al.*, "Modeling of Thermoelectric Effects in Phase Change Memory Cells," *IEEE Trans. Electron Devices*, vol. 61, no. 2, pp. 372–378, 2014, doi: 10.1109/TED.2013.2296305.

[32] A.-K. U. Michel, M. Wuttig, and T. Taubner, "Design Parameters for Phase-Change Materials for Nanostructure Resonance Tuning," *Adv. Opt. Mater.*, vol. 5, no. 18, p. 1700261, Sep. 2017, doi: https://doi.org/10.1002/adom.201700261.

[33] F. Gong, H. M. Duong, and D. V Papavassiliou, "Inter-Carbon Nanotube Contact and Thermal Resistances in Heat Transport of Three-Phase Composites," *J. Phys. Chem. C*, vol. 119, no. 14, pp. 7614–7620, Apr. 2015, doi: 10.1021/acs.jpcc.5b00651.

[34] C. Chassain *et al.*, "Thermal characterization of Ge-rich GST/TiN thin multilayers for phase change memories," *J. Appl. Phys.*, vol. 133, no. 22, p. 225102, Jun. 2023, doi: 10.1063/5.0152049.

[35] S. Gabardi, D. Campi, and M. Bernasconi, "Ab initio calculation of thermal boundary resistance at the interface of metals with GeTe, In3SbTe2 and In2GeTe3 phase change compounds," *J. Comput. Electron.*, vol. 16, no. 4, pp. 1003–1010, 2017, doi: 10.1007/s10825-017-1097-1.

[36] DerChang Kau *et al.*, "A stackable cross point Phase Change Memory," in *2009 IEEE International Electron Devices Meeting (IEDM)*, IEEE, Dec. 2009, pp. 1–4. doi: 10.1109/IEDM.2009.5424263.

[37] W. Wan *et al.*, "A compute-in-memory chip based on resistive random-access memory," *Nature*, vol. 608, no. 7923, pp. 504–512, Aug. 2022, doi: 10.1038/s41586-022-04992-8.

[38] H. C. Jo and W. Y. Choi, "Encapsulation of NEM Memory Switches for Monolithic-Three-Dimensional (M3D) CMOS–NEM Hybrid Circuits," *Micromachines*, vol. 9, no. 7. 2018. doi: 10.3390/mi9070317.

[39] H. H. Radamson *et al.*, "State of the Art and Future Perspectives in Advanced CMOS Technology," *Nanomaterials*, vol. 10, no. 8. 2020. doi: 10.3390/nano10081555.

[40] D. Térébénec *et al.*, "Improvement of Phase-Change Memory Performance by Means of GeTe/Sb2Te3 Superlattices," *Phys. status solidi – Rapid Res. Lett.*, vol. 15, no. 3, p. 2000538, Mar. 2021, doi: https://doi.org/10.1002/pssr.202000538.

[41] C. Yoo *et al.*, "A Review of Advances in Deposition Methods and Material Properties of Superlattice Phase-Change Memory," *ACS Appl. Electron. Mater.*, vol. 5, no. 11, pp. 5794–5808, Nov. 2023, doi: 10.1021/acsaelm.3c01022.

[42] S. Siegert, "Thermal Properties of Phase-Change Materials," RWTH Aachen University. [Online]. Available: https://publications.rwth-aachen.de/record/465219/files/465219.pdf

[43] S. Raoux, "Phase Change Materials," *Annu. Rev. Mater. Res.*, vol. 39, no. 1, pp. 25–48, Jul. 2009, doi: 10.1146/annurev-matsci-082908-145405.
